# L-carnitine infusion does not alleviate lipid-induced insulin resistance and metabolic inflexibility

**DOI:** 10.1371/journal.pone.0239506

**Published:** 2020-09-25

**Authors:** Yvonne M. H. Bruls, Yvo J. M. op den Kamp, Esther Phielix, Lucas Lindeboom, Bas Havekes, Gert Schaart, Esther Moonen-Kornips, Joachim E. Wildberger, Matthijs K. C. Hesselink, Patrick Schrauwen, Vera B. Schrauwen-Hinderling

**Affiliations:** 1 Departments of Radiology and Nuclear Medicine, NUTRIM School for Nutrition and Translational Research in Metabolism, Maastricht University Medical Center, Maastricht, The Netherlands; 2 Departments of Nutrition and Movement Sciences, NUTRIM School for Nutrition and Translational Research in Metabolism, Maastricht University Medical Center, Maastricht, The Netherlands; 3 Division of Endocrinology, Department of Internal Medicine, NUTRIM School for Nutrition and Translational Research in Metabolism, Maastricht University Medical Center, Maastricht, The Netherlands; Garvan Institute of Medical Research, AUSTRALIA

## Abstract

**Background:**

Low carnitine status may underlie the development of insulin resistance and metabolic inflexibility. Intravenous lipid infusion elevates plasma free fatty acid (FFA) concentration and is a model for simulating insulin resistance and metabolic inflexibility in healthy, insulin sensitive volunteers. Here, we hypothesized that co-infusion of L-carnitine may alleviate lipid-induced insulin resistance and metabolic inflexibility.

**Methods:**

In a randomized crossover trial, eight young healthy volunteers underwent hyperinsulinemic-euglycemic clamps (40mU/m^2^/min) with simultaneous infusion of saline (CON), Intralipid (20%, 90mL/h) (LIPID), or Intralipid (20%, 90mL/h) combined with L-carnitine infusion (28mg/kg) (LIPID+CAR). Ten volunteers were randomized for the intervention arms (CON, LIPID and LIPID+CAR), but two dropped-out during the study. Therefore, eight volunteers participated in all three intervention arms and were included for analysis.

**Results:**

L-carnitine infusion elevated plasma free carnitine availability and resulted in a more pronounced increase in plasma acetylcarnitine, short-, medium-, and long-chain acylcarnitines compared to lipid infusion, however no differences in skeletal muscle free carnitine or acetylcarnitine were found. Peripheral insulin sensitivity and metabolic flexibility were blunted upon lipid infusion compared to CON but L-carnitine infusion did not alleviate this.

**Conclusion:**

Acute L-carnitine infusion could not alleviated lipid-induced insulin resistance and metabolic inflexibility and did not alter skeletal muscle carnitine availability. Possibly, lipid-induced insulin resistance may also have affected carnitine uptake and may have blunted the insulin-induced carnitine storage in muscle. Future studies are needed to investigate this.

## Introduction

Type 2 diabetes mellitus is an increasing health problem worldwide. Type 2 diabetes patients and individuals at risk of developing diabetes are characterized by insulin resistance and metabolic inflexibility [1]. The latter is defined as an impaired capacity to switch from lipid oxidation in the fasted state, towards carbohydrate oxidation in the insulin stimulated state [1]. Obesity and excessive availability of lipid substrate are strongly related to insulin resistance and metabolic inflexibility [2]. The infusion of lipids in insulin sensitive subjects temporarily causes lipid-induced insulin resistance and metabolic inflexibility, which is therefore a well-appreciated model to investigate the mechanisms underlying the development of insulin resistance and metabolic inflexibility [3, 4]. It is well known that exercise-trained individuals are more insulin sensitivity and metabolically flexible compared to untrained BMI- and age-matched individuals [4, 5]. Interestingly, when using a lipid infusion model, the degree of lipid induced insulin resistance and metabolic inflexibility differs between exercise-trained and untrained individuals. Exercise-trained athletes remained more insulin sensitive, reflected by a reduction in insulin sensitivity of 29% in compared to 63% in untrained individuals [4]. Why exercise-trained individuals remain more insulin sensitive and metabolic flexible upon lipid infusion is mechanistically still unclear. Possible, the availability of skeletal muscle free carnitine might play a role in here.

Recently, carnitine has been suggested to play a role in maintaining insulin sensitivity and metabolic flexibility [6–8]. Although carnitine is best known for its function in the transport of long-chain fatty acyl-units into the mitochondrial matrix, allowing subsequent β-oxidation [9, 10], it exerts other functions as well. The function of carnitine to conjugate with acetyl-CoA to form acetylcarnitine is gaining increasingly interest as it may be relevant in preserving insulin sensitivity and metabolic flexibility [6]. This conjugation to acetylcarnitine is facilitated by the enzyme carnitine acyl transferase (CrAT) in the mitochondria. With reduced acetylcarnitine formation, acetyl-CoA may accumulate inside mitochondria, especially during conditions of high substrate load (i.e. exercise or high-fat feeding). A rise in acetyl-CoA could inhibit the activity of pyruvate dehydrogenase (PDH), which is a rate-limiting step in the conversion of pyruvate into acetyl-CoA. As a consequence, mitochondria may be less able to maintain high rates of glucose oxidation, which may infer metabolic inflexibility. The availability of carnitine is therefore crucial in acetylcarnitine formation and may be actively involved in maintaining metabolic flexibility, insulin sensitivity and glucose homeostasis [6–8].

Animal studies indeed explored the link between metabolic inflexibility and changes in carnitine status but results are inconclusive [6, 7, 11–13]. While some studies did not reveal a link between metabolic flexibility and changes in carnitine status [12, 13], Noland et al. showed that a reduction in free carnitine availability in rats was associated with decreased metabolic flexibility and that the consumption of a high-fat diet even could lower free carnitine availability [7]. Interestingly, increasing carnitine availability via supplementation in these rats resulted in complete restoration of metabolic flexibility, as well as remaining high PDH activity and consequently insulin sensitivity [7]. Furthermore, observational data showed that obese mice are characterized with low acetylcarnitine concentrations, which could underlie the insulin resistant state [6, 11]. Interestingly, these animals benefit from improved carnitine availability as acetylcarnitine levels restored concomitantly with improved metabolic flexibility, PDH activity, insulin sensitivity, as well as blood glucose levels [11]. The latter suggest better glucose homeostasis and therefore may be a pivotal mechanism to further explore in type 2 diabetes.

In patients with type 2 diabetes, lower skeletal muscle acetylcarnitine concentrations were detected using magnetic resonance spectroscopy [14], which possibly indicate low carnitine availability. Carnitine supplementation has been shown to improve glucose tolerance in insulin resistant subjects with low carnitine status [15]. Some, although not all studies, reported beneficial effects of carnitine administration on plasma glucose, insulin and lactate levels [16–20]. Furthermore, markers of insulin resistance such as glucose infusion rate (GIR) [19, 20] and M-value [21] were reported to improve upon intravenous carnitine administration. However, it still remains elusive what is underlying these beneficial effects of carnitine administration.

We hypothesize that free carnitine availability in skeletal muscle tissue might be crucial in maintaining metabolic flexibility and insulin sensitivity. Especially when lipid availability is increased, free carnitine availability might become limiting. Therefore, in the current study we aimed to investigate if L-carnitine infusion during simultaneous lipid infusion could alleviate lipid-induced insulin resistance and metabolic inflexibility in healthy young males.

## Methods

### Ethical approval

Study procedures were approved by the Medical Ethical Committee in accordance with the declaration of Helsinki. The full name on the ethics committee is the medical-ethical review committee of the academic hospital Maastricht (azM) and Maastricht University (UM), METC azM/UM. Trial monitoring was performed by the Clinical Trial Center Maastricht. The study was registered at clinicaltrials.gov with identifier NCT02722902, full date of first registration was March 30, 2016. All subjects gave written informed consent after the protocol was fully explained.

### General characteristics

Eight young (18–40 years), healthy sedentary lean (BMI: 18–25 kg/m^2^) males participated in this study. Participants were excluded in case of medication use interfering with glucose homeostasis and/or study procedures, exercise engagement exceeding 3 hours a week, smoking, unstable body weight (weight gain or loss > 5kg in the previous 3 months), alcohol and/or drug abuse, impairments in kidney and/or liver function, uncontrolled hypertension, cardiovascular disease. Furthermore, significant food allergies/intolerances to the applied intervention, participation in another biomedical study within 1 month before the first study visit, and anaemia (haemoglobin levels <7.8 mmol/L) were exclusion criteria. Participants who intended to donate blood during the study period or participants who have donated blood less than three months before the start of the study were not included to minimize the risk of anaemia due to repetitive blood sampling in this study. Participants were not included in case they did not wanted their treating physician to be informed about participation in the study. Furthermore, if participants did not want to be informed about unexpected medical findings participation in the study was not possible. Finally, vegetarians were not included because of the altered whole body carnitine status.

### Experimental design

The study was set up as a single blind, placebo-controlled randomized cross-over design. The study was conducted at Maastricht University Medical Center, The Netherlands, between December 2016 and June 2017. Participants were instructed to maintain their usual physical activity patterns and to not change dietary behavior while participating in the study. During visit 1, participants came in the morning after an overnight fast. Body composition (fat percentage and fat free mass) was determined. Subsequently, maximal oxygen uptake (VO_2max_) and maximal power output were determined during an incremental cycling test on a stationary bike to determine training status. On each of the following visits (visit 2, 3 and 4), participants came in at 0600 h after an overnight fast. On each of these visits (visit 2, 3 and 4), a hyperinsulinemic euglycemic clamp was performed to assess peripheral insulin sensitivity. Two hyperinsulinemic euglycemic clamps were performed with simultaneous infusion of lipids. In one of these lipid infusion study arms, subjects received simultaneously L-carnitine infusion (=LIPID + CAR). In the other lipid trial, L-carnitine infusion was replaced by saline infusion (=LIPID). During the third hyperinsulinemic euglycemic clamp, only saline was infused as a control for the lipid infusion (=CON). The sequence of these different hyperinsulinemic-euglycemic clamp conditions was randomly assigned. Block randomization with blocks consisting of three items (1: CON, 2: LIPID, 3: LIPID+CAR) was performed by the researchers as previously described by Snedecor and Cochran [22]. Participants were blinded for treatment. The half-life of release of carnitine from skeletal muscle is 139 hours, therefore a wash-out period of at least two weeks was used to prevent carry over effects of the L-carnitine and lipid infusion. Primary outcome was the effect of additional L-carnitine in combination with lipid infusion on insulin sensitivity and metabolic flexibility compared to only lipid infusion. Secondary outcome measures were plasma and skeletal muscle acylcarnitine profiles.

### Body composition

During the first visit, participants came in after an overnight fast. Body mass and body volume were assessed using air-displacement plethysmography (ADP) using the Bod Pod (Cosmed, Rome, Italy) according to the manufacturer’s instructions and as described previously [23, 24]. Thoracic gas volume was predicted based on equations included in the software. From these data, body composition (fat mass, fat free mass and fat percentage) was calculated as described by Siri [25].

### VO_2_ max

Directly after the body composition determination during the first visit, all participants performed a routine incremental exhaustive cycling test on a stationary bike to determine maximal oxygen uptake (VO_2_max) and maximal power output (W_max_) as reported previously [26] for characterization of the participants and to confirm that the participants were not exercise-trained. Briefly, after a five-minute warming-up period, the workload was increased every 2.5 minutes until exhaustion was reached. Oxygen uptake was measured continuously throughout the test using indirect calorimetry (Omnical, Maastricht, The Netherlands).

### Hyperinsulinemic-euglycemic clamp

At visit 2, 3 and 4, insulin sensitivity was assessed during a 6-hour hyperinsulinemic-euglycemic clamp. Participants refrained from strenuous exercise three days preceding the clamp and monitored their food intake in a food diary. A standardized carbohydrate rich meal was consumed by all participants the evening prior to the clamp. At the day of the clamp, participants reported to university at 0600 h after an overnight fast from 2000 h onwards. A fasted blood sample was obtained and a primed constant 6-hour insulin infusion was started (40mU/m^2^) with simultaneous infusion of variable amounts of glucose (glucose 20%) to maintain euglycemia (5.0mmol/L). Next to the infusion of insulin and glucose, infusion of Intralipid or saline and L-carnitine or saline were started. Intralipid (Fresenius Kabi, Zeist, Nederland) or saline (Braun, Melsungen, Germany) was administrated at an infusion rate of 90ml/h. Intralipid consisted of pure soya-oil including linoleic acid, linolenic acid, oleic acid, palmitic acid and stearic acid. All included lipids are long chain triglycerides (LCT). A primed (4mg/kg) continuous (4mg/kg/h) infusion of L-Carnitine (Carnitene, Alfasigma, Utrecht, The Netherlands) or saline (Braun, Melsungen, Germany) was used. Indirect calorimetry (ventilated hood) was performed and blood samples were taken in the basal state (t = -30-0 min) and the last 30 minutes (t = 330–360 min) of insulin stimulation to determine metabolic flexibility and glucose and lipid oxidation rates according to Peronnet et al. [27]. The respiratory exchange ratio (RER), defined as VCO_2_/VO_2_, was used to determine metabolic flexibility (ΔRER). Metabolic flexibility (ΔRER) reflects the difference between the insulin stimulated RER (t = 330–360 min) minus RER at basal conditions (t = -30-0 min).

### Muscle biopsies

On the day of the hyperinsulinemic-euglycemic clamp (visit 2, 3 and 4), skeletal muscle biopsies were taken upon 6 hours of insulin stimulation. In the control arm, an additional muscle biopsy was taken in the morning after an overnight fast. Muscle biopsies were taken from the *m*. *vastus lateralis* muscle according to the Bergstrom method [28] under local anesthesia (1% Lidocaine, Accord Healthcare Limited, Harrow, United Kingdom). Muscle tissue was immediately frozen in melting isopentane and stored at –80°C until further processing. Skeletal muscle acylcarnitine species were determined using mass spectrometry as described previously [29]. Total short-chain acylcarnitine species included the sum of C3 until C5 carnitine species. C6 until C12 acylcarnitine species were defined as medium-chain acylcarnitine species. Long-chain acylcarnitine species represented C14 until C18-acylcarnitine species.

### Blood sample analysis

During the hyperinsulinemic-euglycemic clamp, the hand was heated in a hot box (55°C) to allow arterialized venous blood sampling from the hand vein. The arterialized venous blood samples were immediately centrifuged and plasma was frozen in liquid nitrogen and stored at -80°C until analyzed. Plasma free fatty acid (FFA) concentrations were determined at t = 120, 180, 240, 360 and 480 minutes via an enzymatic assay automated on a Cobas Dara/Mira analyzer (Wako Nefa C test kit, Wako Chemicals, Neuss, Germany). Plasma acylcarnitine species were measured using tandem mass spectrometry as previously described [30] during the basal and insulin stimulated steady state (t = 120 and t = 480 minutes) and at t = 180 minutes to determine the expected increase in plasma acetylcarnitine levels. Total short-chain acylcarnitine species included the sum of C3 until C5 carnitine species. C6 until C12 acylcarnitine species were defined as medium-chain acylcarnitine species. Long-chain acylcarnitine species represented C14 until C18-acylcarnitine species.

### Sample size calculation and study status

The sample size was calculated based on the results from previous carnitine infusion studies reporting clinically significant improvements in insulin sensitivity after carnitine infusion of 0.5–1.4 mg/kg/min during a hyperinsulinemic-euglycemic clamp [19, 20]. The intraindividual variation (SD) of the difference in insulin sensitivity in repeated measurements is reported to be 0.68 mg/kg/min [31–33]. To reach 80% power, assuming an improvement of 0.5 mg/kg/min, and a significance level of 0.05, a minimal calculated sample size of N = 13 was needed. An interim-analysis was performed after eight participants completed the entire study with all three intervention arms (17 participants were recruited for screening by then), revealing no effect of the carnitine treatment. Therefore, the study was terminated prematurely after eight participants.

### Statistics

The statistical analysis was performed using SPSS 24.0 software (SPSS, Chicago, Il.). All results are presented as mean ± SEM. Statistical significance was set at *P*<0.05. A one-way ANOVA was carried out to investigate differences in insulin sensitivity (M-value), metabolic flexibility (ΔRER) and skeletal muscle acylcarnitine species between study arms. For the comparisons of skeletal muscle acylcarnitine species in the insulin-stimulated states with the basal state, a Student’s paired sample t-test was performed with Bonferroni correction for multiple testing and therefore, p-values of 0.0125 was considered statistically significant. A two-way ANOVA for repeated measures was performed to test differences in GIR, oxidation rates and plasma acylcarnitines. In case of a significant F-value, Bonferroni post-hoc analysis were performed.

## Results

### Participant characteristics

**Fig 1:**
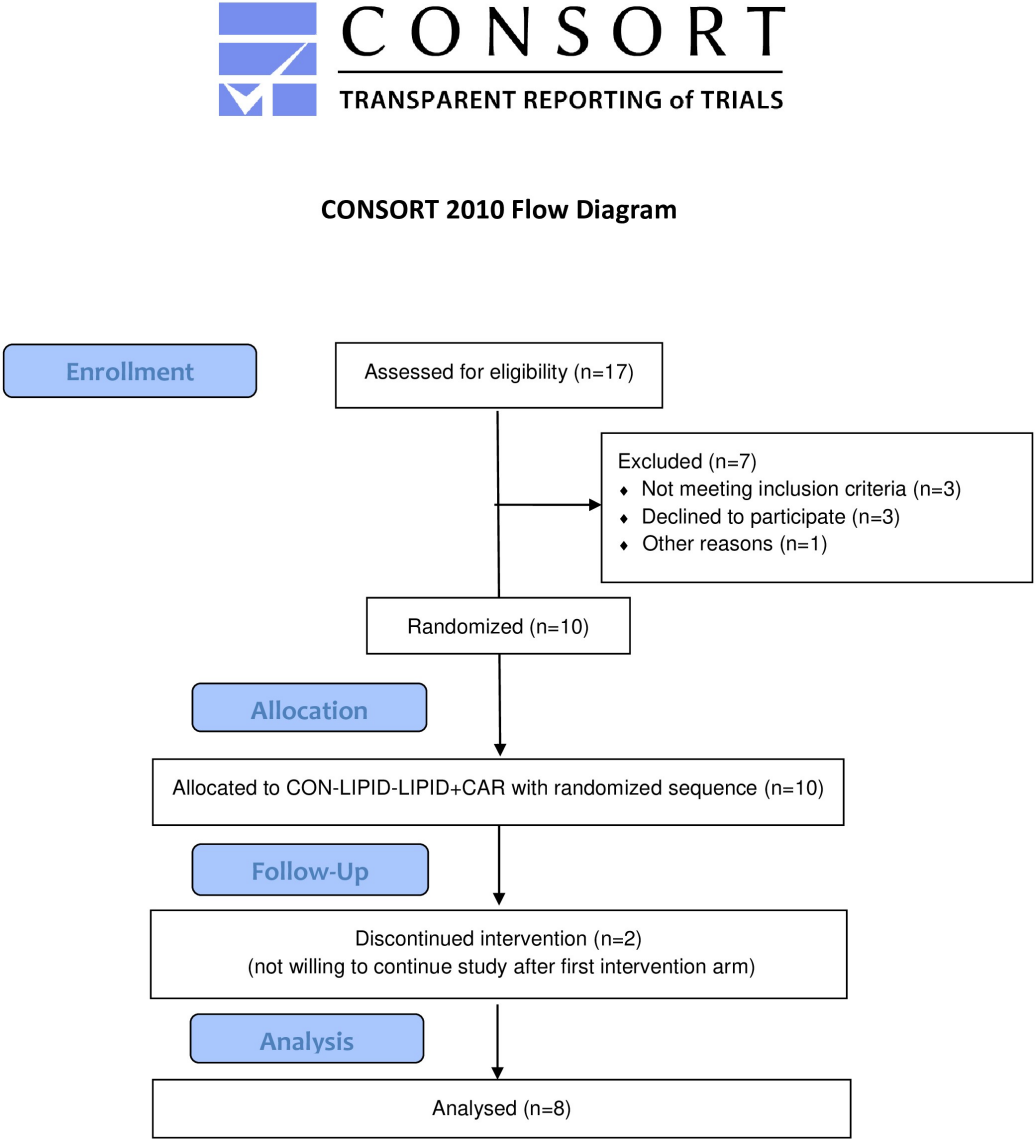
CONSORT flow diagram. Diagram of the progress through the phases of this randomized, controlled crossover study with young lean male participants.

Eight healthy young lean male participants (body weight = 76.5±1.9 kg, BMI = 23.2±0.4 kg/m^2^, age = 22±1 year) were included. No drop-outs were reported. All participants had a sedentary life style (not engaged in regular physical activity). Their maximal oxygen uptake (VO_2_max) (42.4±2.5 ml/min/kg) and body fat percentage (17.1±1.9%) were within the normal range for young, untrained males. Participant enrollment and allocation are presented in Fig 1 whereas characteristics are presented in Table 1. No adverse events of the Intralipid and/or carnitine infusion have occurred in this study.

**Table 1:** Baseline participant characteristics.

	*n* = 8
**Age (y)**	22 ± 1
**Body mass (kg)**	76.5 ± 1.9
**BMI (kg/m^2^)**	23.2 ± 0.4
**Waist-Hip ratio**	0.84 ± 0.02
**Blood pressure**	
**Systolic (mmHg)**	118 ± 2
**Diastolic (mmHg)**	72 ± 1
**Body composition**	
**Fat mass (kg)**	12.9 ± 1.6
**Fat free mass (kg)**	61.3 ± 2.1
**Fat percentage (%)**	17.1 ± 1.9
**Physical fitness**	
VO_2_max (ml*min^-1^*kg bw^-1^)	42.4 ± 2.5
Wmax (W*kgbw^-1^)	3.3 ± 0.2
**Glucose metabolism**	
**Fasting glucose (mmol/L)**	4.9 ± 0.1
**Fasting insulin (pmol/L)**	28.9 ± 3.9
**HbA1c (%)**	5.2 ± 0.1
**Blood lipid profile**	
**Total cholesterol (mmol/L)**	4.1 ± 0.2
**HDL cholesterol (mmol/L)**	1.5 ± 0.1
**LDL cholesterol (mmol/L)**	2.2 ± 0.1
**Triglycerides (mmol/L)**	1.0 ± 0.1

Data are represented as mean ± S.E.M. Wmax, maximal workload, VO_2_max is normalized to body weight in kg

### Insulin sensitivity

**Fig 2:**
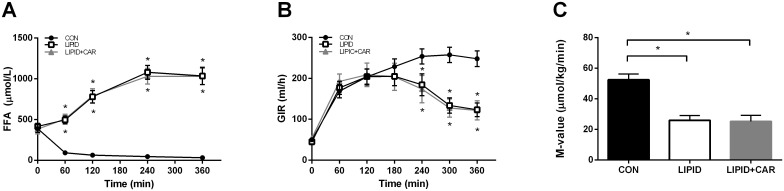
Hyperinsulinemic-euglycemic clamp. Plasma FFA concentrations (A), glucose infusion rates (B) and insulin sensitivity expressed as M-value (C) in CON, LIPID and LIPID+CAR during the hyperinsulinemic-euglycemic clamp (n = 8). Data are expressed as mean + SEM. *, Significantly different from CON (*P*<0.05).

At baseline, plasma FFA levels were comparable between study arms (389±47 vs. 415±40 vs. 382±60 μmol/L in CON, LIPID and LIPID+CAR respectively, *P* = 0.885). In the lipid trial an increase in FFA levels occurred over time and were significantly higher at all time points compared to the control condition (*P* <0.01, Fig 2A). Simultaneous infusion of L-carnitine did not alter FFA levels when compared to lipid infusion alone (*P* = 0.939, Fig 2A).

During the first three hours of the clamp, glucose infusion rates (GIR) were comparable between all three study arms (*P* = 0.448 Fig 2B). From 3 hours onwards, glucose infusion rates were lower in LIPID as well as in LIPID+CAR compared to CON (*P*<0.01, Fig 2B), consistent with previous studies showing that lipid-induced insulin resistance occurs after 2–3 hours of lipid infusion [4, 34]. No difference was found in GIR at any time point between LIPID and LIPID+CAR (*P* = 0.897) indicating that L-carnitine infusion did not alter lipid-induced insulin resistance (Fig 2B).

As a result, peripheral insulin sensitivity, expressed as the M-value, was blunted during the lipid infusion compared to the control condition (26.0±3.1 vs. 52.5±3.8 μmol/kg/min, *P* = 0.019 respectively). Lipid-induced insulin resistance was not alleviated by L-carnitine infusion (M-value LIPID+CAR; 25.3±4.0 μmol/kg/min, *P*>0.99 compared to LIPID, Fig 2C).

### Metabolic flexibility and substrate oxidation

**Fig 3:**
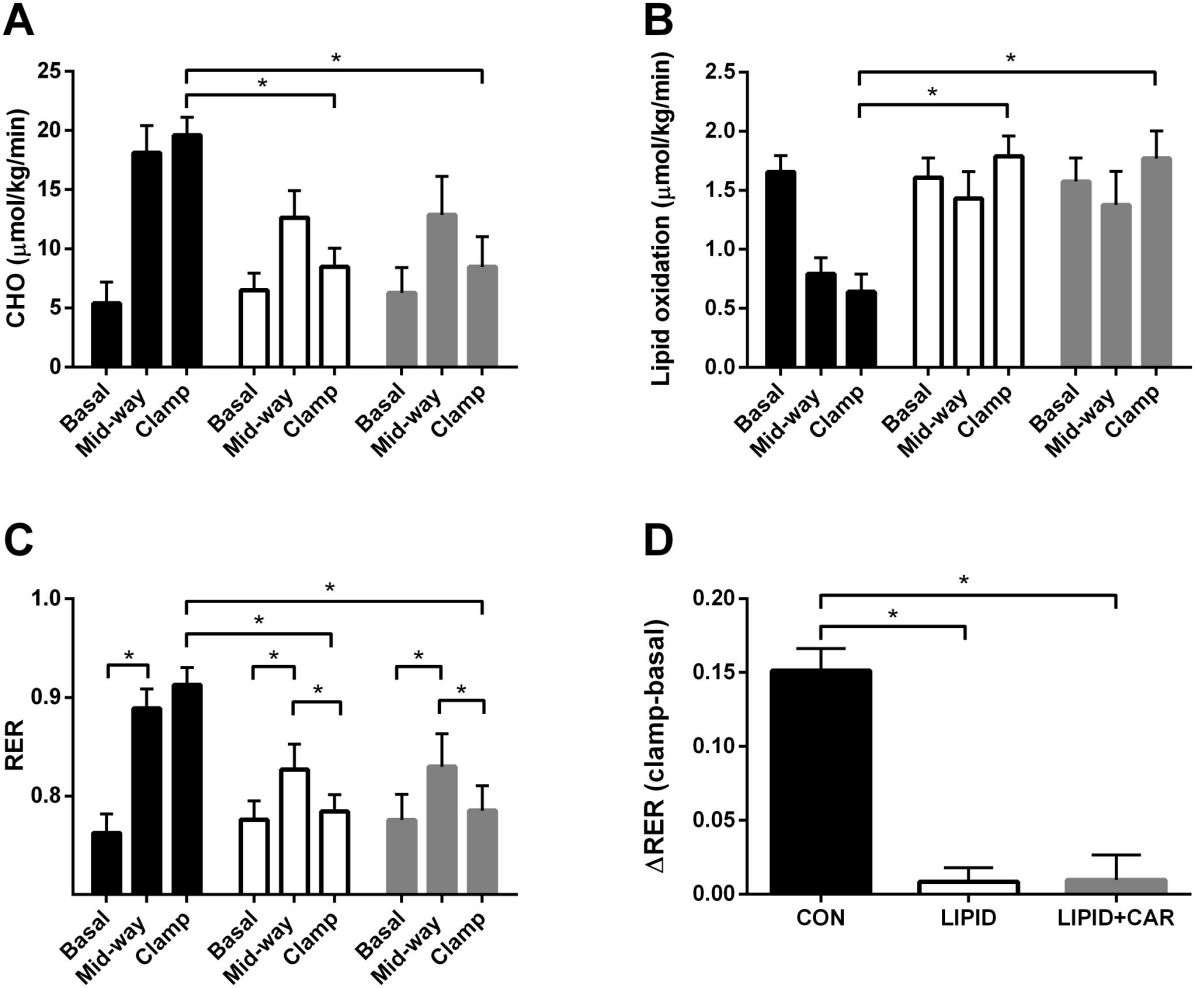
Metabolic flexibility and substrate oxidation. Glucose oxidation (A), Lipid oxidation (B), respiratory exchange ratio (RER) (C) and metabolic flexibility expressed as delta RER (D) assessed during a hyperinsulinemic-euglycemic clamp in CON, LIPID and LIPID+CAR (n = 8). Black bars represent CON, white bars LIPID and grey bars represent LIPID+CAR. Basal clamp is measured from t = -30-t = 0, mid-way from t = 120–150 and clamp from t = 330–360. Data are expressed as mean + SEM. *, Significantly different from CON (*P*<0.05).

Basal glucose oxidation were comparable between study arms but glucose oxidation upon 6 hours of insulin infusion was increased in the control condition (from 5.4±1.8 to 19.6±1.5 μmol/kg/min, *P*<0.01, Fig 3A). However, glucose oxidation remained low during the infusion of lipid (from 6.5±1.5 to 8.5±1.6 μmol/kg/min, *P* = 0.164, Fig 3A). Parallel infusion of L-carnitine did not change the lipid-induced suppression in glucose oxidation (*P* = 0.864). In line with these findings, lipid oxidation was comparable between study arms at baseline but was elevated after 6-hours of lipid infusion and suppressed in the control arm (1.8±2.7 and 0.6±0.2 μmol/kg/min in LIPID and CON respectively, *P*<0.01, Fig 3B). Also here, L-carnitine infusion did not affect lipid oxidation rates (*P* = 0.883, Table 2).

**Table 2:** Substrate kinetics and insulin sensitivity.

	CON	LIPID	LIPID+CAR
**RER (Arbitrary Units)**			
**Basal (t = -30-0)**	0.78 ± 0.02	0.78 ± 0.02	0.78 ± 0.03
**Middle (t = 120–150)**	0.90 ± 0.02	0.83 ± 0.03	0.83 ± 0.03
**Insulin stimulated (t = 330–360)**	0.91 ± 0.02	0.78 ± 0.02^a^	0.79 ± 0.03^a^
**Δ _clamp-basal_**	0.10 ± 0.02	0.01 ± 0.01^a^	0.01 ± 0.01^a^
CHO oxidation (μmol*kg^-1^*min^-1^)			
**Basal (t = -30-0)**	5.4 ± 1.8	6.5 ± 1.5	6.3 ± 2.2
**Middle (t = 120–150)**	18.1 ± 2.3	12.6 ± 2.3	12.8 ± 3.3
**Insulin stimulated (t = 330–360)**	19.6 ± 1.5	8.5 ± 1.6^a^	8.5 ± 2.6^a^
**Δ _clamp-basal_**	9.5 ± 2.2	1.4 ± 0.8^a^	1.6 ± 1.2^a^
Lipid oxidation (μmol*kg^-1^*min^-1^)			
**Basal (t = -30-0)**	1.7 ± 0.1	1.6 ±0.2	1.6 ± 0.2
**Middle (t = 120–150)**	0.8 ± 0.1	1.4 ± 0.2	1.4 ± 0.3
**Insulin stimulated (t = 330–360)**	0.6 ± 0.2	1.8 ± 0.2^a^	1.8 ± 0.2^a^
**Δ _clamp-basal_**	-1.01 ± 0.11	0.18 ± 0.08^a^	0.19 ± 0.15^a^
M-value (μmol*kg^-1^*min^-1^)	52.5 ± 3.8	26.0 ± 3.1^a^	25.3 ± 4.0^a^

Data are expressed as mean ± S.E.M. (*n* = 8).

^*a*^ significantly different from CON.

The RER measured under baseline conditions, so before the start of the infusions, was not different between study arms, as expected (*P* = 0.881, Fig 3C). Metabolic flexibility, expressed as ΔRER_clamp-basal_, was decreased upon lipid infusion compared to control (0.10±0.02 and 0.01±0.01 in CON and LIPID respectively, *P*<0.01, Fig 3D). L-carnitine did not change the lipid-induced decrease in metabolic flexibility (0.01±0.01 in LIPID+CAR, *P* = 0.920).

### Plasma acylcarnitine profiles

**Fig 4:**
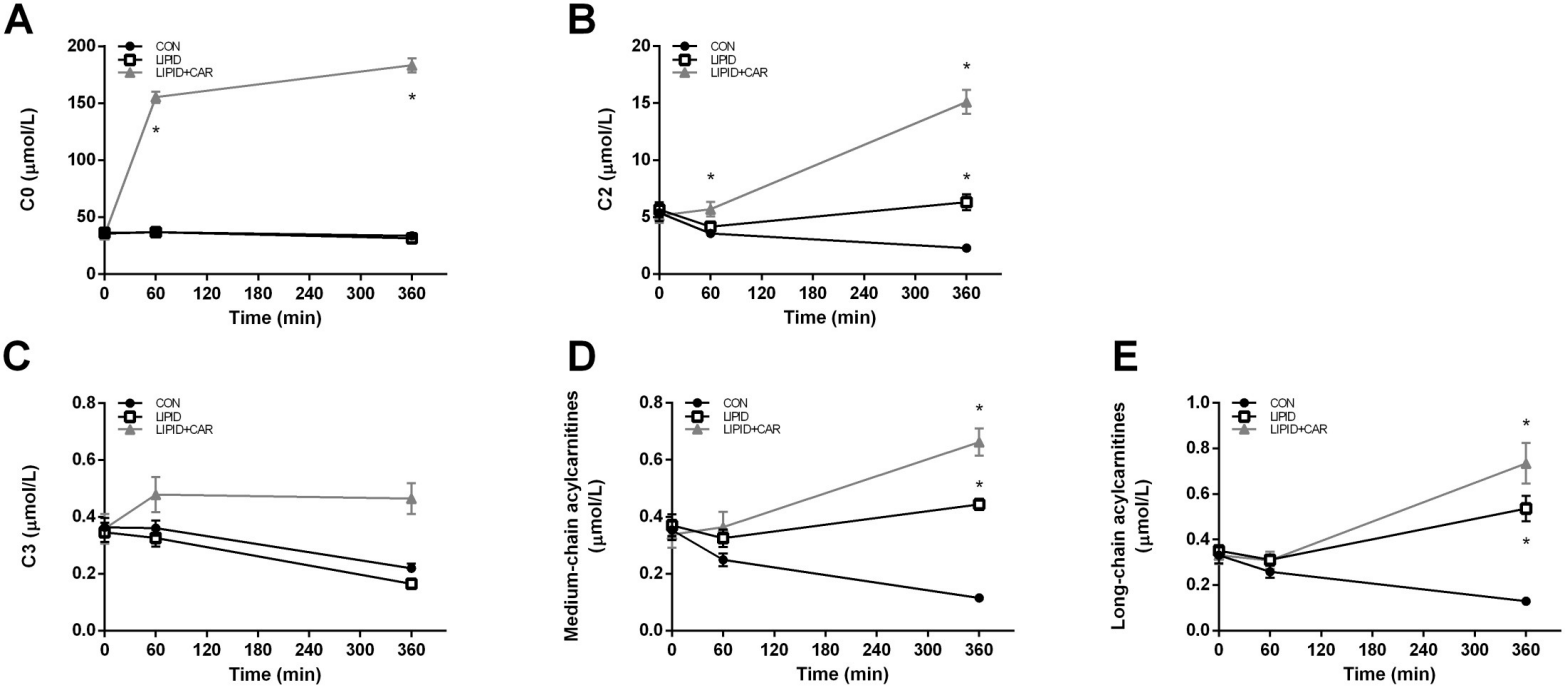
Plasma acylcarnitine profiles. Plasma free carnitine availability (A), acetylcarnitine (B), C3 acylcarnitine (C), medium chain acylcarnitines (D) and long-chain acylcarnitine concentrations (E) measured at baseline (t = 0), after one hour (t = 60) and at the of the 6-hour hyperinsulinemic-euglycemic clamp (t = 360) (n = 8). Black dots represent the control group, white dots the LIPID group and light grey lines the LIPID+CAR group. Data are expressed as means ± SEM. * significantly different from CON (*P*<0.05). Note: in A the lines of the control and lipid conditions are overlapping.

A time*treatment interaction was present for plasma free carnitine availability (*P*<0.01). Plasma free carnitine levels were similar at baseline (35.8±2.0 vs. 36.3±2.8 vs. 34.4±1.9 μmol/L in CON, LIPID, LIPID+CAR respectively, *P* = 0.829, Fig 4A and Table 3). One hour of L-carnitine infusion already increased plasma free carnitine availability to supra-physiological concentrations (155±5 μmol/L, *P*<0.01) and finally reaching concentrations of 183±6 μmol/L (*P*<0.01) after six hours of infusion (Fig 4A and Table 3). No changes from baseline in plasma free carnitine availability were observed in the CON and LIPID trial over time (*P*>0.99, Fig 4A and Table 3).

**Table 3:** Plasma acylcarnitine concentrations before, during and after the 6-hour hyperinsulinemic-euglycemic clamp.

		CON	LIPID	LIPID+CAR
**C0**	t = 0	35.79 ± 2.02	36.32 ± 2.82	34.40 ± 1.87
	t = 60	36.84 ± 2.09	36.96 ± 2.64	155.38 ± 4.94^a^^,^^b^^,^^c^
	t = 360	33.78 ± 2.04	31.55 ± 2.16	183.33 ± 6.28^a^^,^^b^^,^^c^^,^^d^
**C2**	t = 0	5.38 ± 0.71	5.65 ± 0.65	5.13 ± 0.65
	t = 60	3.56 ± 0.34^c^	4.16 ± 0.37^c^	5.70 ± 0.62^a^^,^^b^
	t = 360	2.27 ± 0.10^c^^,^^d^	6.31 ± 0.69^a^^,^^d^	15.11 ± 1.04^a^^,^^b^^,^^c^^,^^d^
**C3**	t = 0	0.36 ± 0.03	0.35 ± 0.03	0.36 ± 0.05
	t = 60	0.36 ± 0.03	0.33 ± 0.03	0.48 ± 0.06^a^^,^^b^^,^^c^
	t = 360	0.22 ± 0.02^c^^,^^d^	0.17 ± 0.01^a^^,^^c^^,^^d^	0.47 ± 0.05^a^^,^^b^^,^^c^
**C4**	t = 0	0.20 ± 0.01	0.18 ± 0.01	0.20 ± 0.02
	t = 60	0.19 ± 0.02	0.17 ± 0.01	0.22 ± 0.02^b^^,^^c^
	t = 360	0.15 ± 0.01^c^^,^^d^	0.14 ± 0.01^c^^,^^d^	0.28 ± 0.03^a^^,^^b^^,^^c^^,^^d^
**C5:1**	t = 0	0.01 ± 0.00	0.01 ± 0.00	0.01 ± 0.00
	t = 60	0.01 ± 0.00	0.01 ± 0.00	0.01 ± 0.00
	t = 360	0.00 ± 0.00	0.00 ± 0.00^c^	0.01 ± 0.00
**C5**	t = 0	0.09 ± 0.01	0.09 ± 0.01	0.09 ± 0.01
	t = 60	0.09 ± 0.01	0.09 ± 0.01	0.11 ± 0.01^a^^,^^b^^,^^c^
	t = 360	0.05 ± 0.01^c^^,^^d^	0.04 ± 0.01^c^^,^^d^	0.06 ± 0.01^b^^,^^c^^,^^d^
**C4-3OH**	t = 0	0.03 ± 0.00	0.04 ± 0.01	0.04 ± 0.01
	t = 60	0.02 ± 0.00^c^	0.03 ± 0.00^c^	0.05 ± 0.02
	t = 360	0.01 ± 0.00^c^^,^^d^	0.06 ± 0.01^a^^,^^c^^,^^d^	0.14 ± 0.03^a^^,^^b^^,^^c^^,^^d^
**C6**	t = 0	0.04 ± 0.00	0.04 ± 0.00	0.03 ± 0.00
	t = 60	0.03 ± 0.00^c^	0.03 ± 0.00^c^	0.04 ± 0.01^a^
	t = 360	0.02 ± 0.00^c^^,^^d^	0.04 ± 0.00^a^^,^^c^^,^^d^	0.08 ± 0.01^a^^,^^b^^,^^c^^,^^d^
**C5OH**	t = 0	0.01 ± 0.00	0.02 ± 0.00	0.01 ± 0.00
	t = 60	0.01 ± 0.00	0.02 ± 0.00	0.02 ± 0.00
	t = 360	0.00 ± 0.00	0.01 ± 0.00^d^	0.01 ± 0.00
**C8**	t = 0	0.09 ± 0.01	0.09 ± 0.01	0.08 ± 0.01
	t = 60	0.06 ± 0.00^c^	0.07 ± 0.01^c^	0.08 ± 0.01
	t = 360	0.03 ± 0.00^c^^,^^d^	0.07 ± 0.01^a^	0.10 ± 0.01^a^^,^^b^^,^^c^^,^^d^
**C3 DC**	t = 0	0.03 ± 0.00	0.04 ± 0.00	0.04 ± 0.01
	t = 60	0.03 ± 0.00^c^	0.03 ± 0.00^a^^,^^c^	0.03 ± 0.00^c^
	t = 360	0.01 ± 0.00^c^^,^^d^	0.03 ± 0.00^a^	0.04 ± 0.00^a^^,^^d^
**C10:1**	t = 0	0.06 ± 0.01	0.06 ± 0.01	0.05 ± 0.01
	t = 60	0.04 ± 0.01^c^	0.09 ± 0.01^a^^,^^c^	0.10 ± 0.02^a^^,^^c^
	t = 360	0.01 ± 0.00^c^^,^^d^	0.19 ± 0.01^a^^,^^c^^,^^d^	0.27 ± 0.02^a^^,^^b^^,^^c^^,^^d^
**C10**	t = 0	0.07 ± 0.01	0.08 ± 0.01	0.07 ± 0.01^c^
	t = 60	0.05 ± 0.01^c^	0.05 ± 0.01^c^	0.06 ± 0.01
	t = 360	0.01 ± 0.00^c^^,^^d^	0.04 ± 0.00^a^^,^^c^	0.06 ± 0.01^a^^,^^b^
**C4 DC**	t = 0	0.04 ± 0.00	0.04 ± 0.01	0.03 ± 0.01
	t = 60	0.04 ± 0.00	0.04 ± 0.01	0.03 ± 0.01
	t = 360	0.04 ± 0.00^c^	0.04 ± 0.00	0.03 ± 0.00
**C5 DC**	t = 0	0.03 ± 0.00	0.03 ± 0.00	0.03 ± 0.00
	t = 60	0.02 ± 0.00	0.02 ± 0.00	0.03 ± 0.00^a^
	t = 360	0.02 ± 0.00^c^	0.02 ± 0.00^a^	0.03 ± 0.00^a^
**C12:1**	t = 0	0.03 ± 0.00	0.03 ± 0.00	0.03 ± 0.01
	t = 60	0.02 ± 0.00	0.02 ± 0.00^c^	0.02 ± 0.00^c^
	t = 360	0.00 ± 0.00^c^^,^^d^	0.03 ± 0.00^a^^,^^d^	0.05 ± 0.00^a^^,^^b^^,^^d^
**C12**	t = 0	0.03 ± 0.00	0.03 ± 0.00	0.03 ± 0.01
	t = 60	0.02 ± 0.00^c^	0.02 ± 0.00^c^	0.03 ± 0.00^c^
	t = 360	0.01 ± 0.00^c^^,^^d^	0.02 ± 0.00^a^^,^^c^	0.03 ± 0.00^a^^,^^b^^,^^d^
**C6 DC**	t = 0	0.01 ± 0.00	0.02 ± 0.00	0.01 ± 0.00
	t = 60	0.01 ± 0.00	0.01 ± 0.00	0.01 ± 0.00
	t = 360	0.01 ± 0.00	0.02 ± 0.00^a^	0.02 ± 0.00^a^^,^^b^^,^^c^^,^^d^
**C12:1OH**	t = 0	0.01 ± 0.00	0.01 ± 0.00	0.01 ± 0.00
	t = 60	0.01 ± 0.00	0.01 ± 0.00	0.01 ± 0.00
	t = 360	0.01 ± 0.00	0.01 ± 0.00^a^	0.02 ± 0.00^a^^,^^b^^,^^c^^,^^d^
**C12OH**	t = 0	0.01 ± 0.00	0.01 ± 0.00	0.01 ± 0.00
	t = 60	0.01 ± 0.00	0.01 ± 0.00	0.01 ± 0.00
	t = 360	0.01 ± 0.00	0.01 ± 0.00	0.01 ± 0.00
**C53M3OH DC**	t = 0	0.00 ± 0.00	0.00 ± 0.00	0.00 ± 0.00
	t = 60	0.00 ± 0.00	0.00 ± 0.00	0.00 ± 0.00
	t = 360	0.00 ± 0.00	0.00 ± 0.00	0.00 ± 0.00
**C14:2**	t = 0	0.02 ± 0.00	0.02 ± 0.00	0.02 ± 0.00
	t = 60	0.01 ± 0.00^c^	0.03 ± 0.00^a^^,^^c^	0.03 ± 0.00^a^^,^^c^
	t = 360	0.00 ± 0.00^c^^,^^d^	0.12 ± 0.02^a^^,^^c^^,^^d^	0.21 ± 0.04^a^^,^^b^^,^^c^^,^^d^
**C14:1**	t = 0	0.04 ± 0.01	0.04 ± 0.01	0.04 ± 0.01
	t = 60	0.03 ± 0.00^c^	0.03 ± 0.00^c^	0.03 ± 0.01
	t = 360	0.01 ± 0.00^c^^,^^d^	0.06 ± 0.01^a^^,^^d^	0.10 ± 0.02^a^^,^^b^^,^^c^^,^^d^
**C14**	t = 0	0.02 ± 0.00	0.02 ± 0.00	0.02 ± 0.00
	t = 60	0.02 ± 0.00	0.01 ± 0.00^c^	0.01 ± 0.00^c^
	t = 360	0.01 ± 0.00^c^^,^^d^	0.01 ± 0.00^a^^.^^c^	0.02 ± 0.00^a^^,^^b^
**C8 DC**	t = 0	0.00 ± 0.00	0.00 ± 0.00	0.00 ± 0.00
	t = 60	0.00 ± 0.00	0.00 ± 0.00	0.00 ± 0.00
	t = 360	0.00 ± 0.00	0.01 ± 0.00^a^^,^^c^^,^^d^	0.01 ± 0.00^a^^,^^c^^,^^d^
**C14:1OH**	t = 0	0.01 ± 0.00	0.01 ± 0.00	0.01 ± 0.00
	t = 60	0.01 ± 0.00	0.01 ± 0.00	0.01 ± 0.00
	t = 360	0.01 ± 0.00	0.01 ± 0.00^a^	0.01 ± 0.00^a^
**C14OH**	t = 0	0.00 ± 0.00	0.01 ± 0.00	0.00 ± 0.00
	t = 60	0.00 ± 0.00	0.00 ± 0.00^c^	0.00 ± 0.00
	t = 360	0.00 ± 0.00	0.00 ± 0.00^c^	0.00 ± 0.00
**C16:1**	t = 0	0.02 ± 0.00	0.02 ± 0.00	0.02 ± 0.00
	t = 60	0.01 ± 0.00^c^	0.01 ± 0.00^c^	0.01 ± 0.00
	t = 360	0.00 ± 0.00^c^^,^^d^	0.02 ± 0.00^a^	0.02 ± 0.00^a^
**C16**	t = 0	0.08 ± 0.01	0.08 ± 0.00	0.08 ± 0.01
	t = 60	0.07 ± 0.01^c^	0.08 ± 0.00	0.07 ± 0.01^c^
	t = 360	0.03 ± 0.00^c^^,^^d^	0.07 ± 0.00^a^^,^^c^^,^^d^	0.08 ± 0.01^a^^,^^b^
**C10 DC**	t = 0	0.00 ± 0.00	0.00 ± 0.00	0.00 ± 0.00
	t = 60	0.00 ± 0.00	0.01 ± 0.00	0.01 ± 0.00
	t = 360	0.00 ± 0.00	0.00 ± 0.00	0.01 ± 0.00
**C16:1OH**	t = 0	0.00 ± 0.00	0.00 ± 0.00	0.00 ± 0.00
	t = 60	0.00 ± 0.00	0.00 ± 0.00	0.00 ± 0.00
	t = 360	0.00 ± 0.00	0.00 ± 0.00	0.00 ± 0.00
**C16OH**	t = 0	0.00 ± 0.00	0.00 ± 0.00	0.00 ± 0.00
	t = 60	0.00 ± 0.00	0.00 ± 0.00	0.00 ± 0.00
	t = 360	0.00 ± 0.00	0.00 ± 0.00	0.00 ± 0.00
**C18:2**	t = 0	0.03 ± 0.00	0.03 ± 0.00	0.03 ± 0.00
	t = 60	0.03 ± 0.00^c^	0.04 ± 0.00^a^^,^^c^	0.04 ± 0.00^a^^,^^c^
	t = 360	0.02 ± 0.00^c^^,^^d^	0.13 ± 0.01^a^^,^^c^^,^^d^	0.15 ± 0.02^a^^,^^b^^,^^c^^,^^d^
**C18:1**	t = 0	0.08 ± 0.01	0.10 ± 0.01	0.09 ± 0.01
	t = 60	0.06 ± 0.01^c^	0.07 ± 0.01^c^	0.07 ± 0.01^c^
	t = 360	0.04 ± 0.00^c^^,^^d^	0.08 ± 0.01^a^	0.09 ± 0.00^a^^,^^b^^,^^c^^,^^d^
**C18**	t = 0	0.03 ± 0.00	0.03 ± 0.00	0.03 ± 0.00
	t = 60	0.03 ± 0.00	0.03 ± 0.00	0.03 ± 0.00
	t = 360	0.02 ± 0.00^c^^,^^d^	0.03 ± 0.00^a^	0.03 ± 0.00^a^
**C18:2OH**	t = 0	0.00 ± 0.00	0.00 ± 0.00	0.00 ± 0.00
	t = 60	0.00 ± 0.00	0.00 ± 0.00	0.00 ± 0.00^c^^,^^d^
	t = 360	0.00 ± 0.00	0.01 ± 0.00^a^^,^^c^	0.01 ± 0.00^a^
**C18:1OH**	t = 0	0.00 ± 0.00	0.00 ± 0.00	0.00 ± 0.00
	t = 60	0.00 ± 0.00	0.00 ± 0.00	0.00 ± 0.00
	t = 360	0.00 ± 0.00	0.00 ± 0.00	0.01 ± 0.00^a^^,^^c^^,^^d^
**C18OH**	t = 0	0.00 ± 0.00	0.00 ± 0.00	0.00 ± 0.00
	t = 60	0.00 ± 0.00	0.00 ± 0.00	0.00 ± 0.00
	t = 360	0.00 ± 0.00	0.00 ± 0.00	0.00 ± 0.00
**Short-**	t = 0	0.80 ± 0.06	0.78 ± 0.06	0.79 ± 0.07
**Chain**	t = 60	0.76 ± 0.05	0.72 ± 0.05	0.97 ± 0.08^a^^,^^b^^,^^c^
	t = 360	0.50 ± 0.04^c^^,^^d^	0.51 ± 0.03^c^^,^^d^	1.06 ± 0.08^a^^,^^b^^,^^c^
**Medium-**	t = 0	0.35 ± 0.04	0.37 ± 0.04	0.34 ± 0.05
**Chain**	t = 60	0.25 ± 0.02^c^	0.33 ± 0.03^a^	0.36 ± 0.05^a^
	t = 360	0.12 ± 0.00^c^^,^^d^	0.44 ± 0.02^c^^,^^d^	0.66 ± 0.05^c^^,^^d^
**Long-**	t = 0	0.33 ± 0.04	0.35 ± 0.02	0.33 ± 0.04
**Chain**	t = 60	0.26 ± 0.03^c^	0.31 ± 0.02^a^^,^^c^	0.31 ± 0.04^a^
	t = 360	0.13 ± 0.01^c^^,^^d^	0.53 ± 0.05^a^^,^^c^^,^^d^	0.74 ± 0.09^a^^,^^b^^,^^c^^,^^d^

Data are expressed as mean ± SEM.

^*a*^ significantly different from CON.

^b^ significantly different from LIPID.

^c^ significantly different from t = 0.

^d^ significantly different from t = 60.

Plasma acetylcarnitine (C2) concentrations showed a time*treatment interaction (*P*<0.01) and were comparable between study arms at baseline (*P* = 0.860). C2 concentrations decreased in the CON trial over time from 5.4±0.7 to 3.6±0 μmol/L after one hour and to 2.3±0.1 μmol/L after six hours (*P*<0.01, Fig 4B and Table 3), which is probably due to insulin infusion. With lipid infusion, C2 concentrations decreased during the first hour (5.7±0.6 to 4.2±0.4 μmol/L, *P*<0.01) and subsequently tended to increase again (*P* = 0.05 compared to t = 60), reaching concentrations of 6.3±0.7 μmol/L after 6 hours which are comparable to baseline values (*P* = 0.375). Infusion of L-carnitine in addition to lipids prevented the decrease in C2 concentrations after one hour resulting in significantly higher C2 levels compared to CON and LIPID. After six hours, plasma acetylcarnitine concentrations were increased compared to baseline (*P*<0.01, Fig 4B and Table 3).

C3 and short-chain acylcarnitines (C3 until C5) both showed a time*treatment interaction (*P*<0.01) and were similar between groups in the basal state after an overnight fast. C3 and short-chain acylcarnitine decreased in the CON and LIPID condition after six hours (*P*<0.01), but were increased upon 6 hours L-carnitine infusion compared to the CON and LIPID condition (*P*<0.01, Fig 4C and Table 3).

A time*treatment interaction was found for both medium- and long-chain acylcarnitines (*P*<0.01). Plasma medium and long-chain acylcarnitines were not different between groups in the basal state after an overnight fast. Insulin stimulation resulted in a reduction in medium- as well as long-chain acylcarnitines in the control group in time (*P*<0.01). In contrast, medium- and long-chain acylcarnitines increased upon lipid infusion after six hours (*P* = 0.034 and *P* = 0.013 respectively, Fig 4D and 4E and Table 3). This increase was even more pronounced when combining lipid infusion with L-carnitine infusion for both medium- as long-chain acylcarnitines (*P*<0.01, Fig 4D and 4E and Table 3).

### Skeletal muscle acylcarnitines profiles

**Fig 5:**
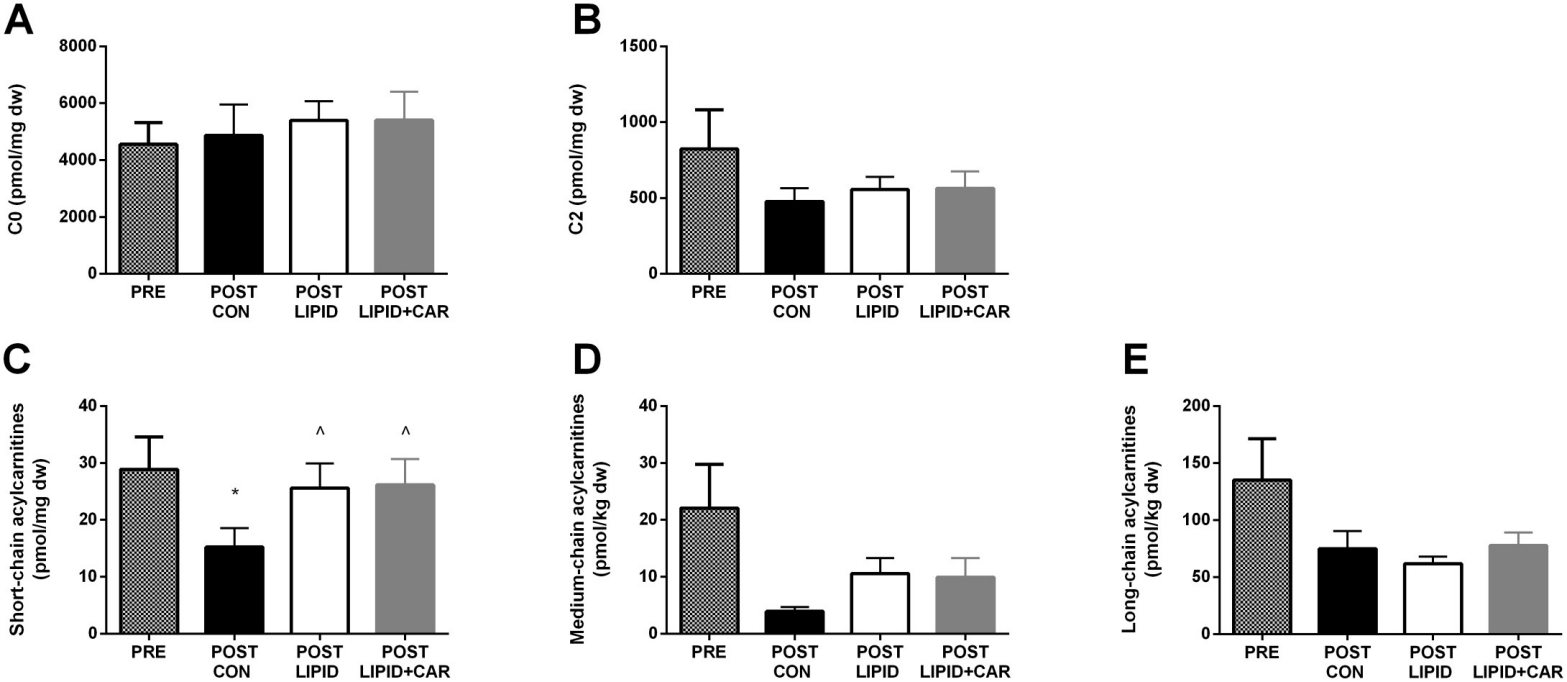
Skeletal muscle acylcarnitine profiles. Skeletal muscle acylcarnitine concentrations measured in biopsies at the end of the 6-hour hyperinsulinemic-euglycemic clamp (n = 8). Black bars represent the control group, white bars the LIPID group and light grey bars the LIPID+CAR group. Dark grey bars represent the pre-clamp muscle biopsy. Data are expressed as means ± SEM. * significantly different from PRE (*P*<0.0125 after Bonferroni correction for multiple testing), ^ tending to be different from CON (*P*<0.10).

No differences in skeletal muscle free carnitine availability (*P* = 0.901) and acetylcarnitine concentrations (*P* = 0.786) were found after 6-hours of infusion between groups (Fig 5A and 5B, Table 4). Because of Bonferroni correction for multiple testing, p-values of 0.0125 were considered statistically significant for the comparison of insulin-stimulated states with the basal state. Free carnitine and acetylcarnitine values obtained after 6 hours were not different from baseline (C0: *P* = 0.734 (CON), *P* = 0.170 (LIPID), *P* = 0.192 (LIPID+CAR) and C2: *P* = 0.138 (CON), *P* = 0.368 (LIPID), *P* = 0.187 (LIPID+CAR)). Short-chain acylcarnitine species reduced upon 6-hours of insulin infusion in CON trial compared to basal (*P* = 0.013). This decrease was blunted upon LIPID and LIPID+CARN compared to CON, resulting in a tendency towards higher short-chain acylcarnitine concentrations upon LIPID and LIPID+CARN compared to CON (*P* = 0.103, Fig 5C and Table 4). Medium and long-chain acylcarnitines seemed to be decreased in CON compared to baseline after 6 hours of insulin infusion, but this did not reach significance (*P* = 0.049 and *P* = 0.107 respectively, Fig 5D and 5E). No difference in medium and long-chain acylcarnitines were found after 6-hours of infusion between groups (*P* = 0.177 and *P* = 0.564 respectively, Fig 5D and 5E and Table 4).

**Table 4:** Skeletal muscle acylcarnitine concentrations (in biopsies) before (only LIPID+CAR trial) and after the 6-hour hyperinsulinemic-euglycemic clamp.

	CON after	LIPID after	LIPID+CAR before	LIPID+CAR after
**C0**	4865.56 ± 1081.90	5391.99 ± 685.81	4559.25 ± 761.19	5402.57 ± 998.28
**C2**	476.28 ± 89.07	556.02 ± 83.27	822.57 ± 257.62	564.47 ± 111.55
**C3**	6.71 ± 1.43	6.84 ± 0.82	8.06 ± 1.30	7.15 ± 0.98
**C4**	2.86 ± 0.74	5.26 ± 1.02	8.55 ± 1.94	4.96 ± 1.44
**C5:1**	0.83 ± 0.33	0.63 ± 0.15	0.64 ± 0.23	0.43 ± 0.13
**C5**	2.59 ± 0.76	1.89 ± 0.40	3.78 ± 1.08	1.74 ± 0.45
**C4-3OH**	1.14 ± 0.20	9.56 ± 2.18^a^	6.26 ± 2.80	10.39 ± 2.90^a^
**C6**	0.79 ± 0.21	3.76 ± 1.29	8.15 ± 3.08	3.58 ± 1.58
**C8:1**	0.44 ± 0.13	1.79 ± 0.33^a^^c^	0.77 ± 0.18	1.18 ± 0.27
**C8**	0.66 ± 0.13	2.30 ± 0.81	4.47 ± 1.60	2.22 ± 0.84
**C4DC**	1.12 ± 0.13	1.45 ± 0.17	1.59 ± 0.29	1.48 ± 0.25
**C10**	0.51 ± 0.11	0.95 ± 0.24	2.70 ± 0.87	1.03 ± 0.32
**C12:1**	0.30 ± 0.07	0.44 ± 0.11	1.20 ± 0.45	0.52 ± 0.14
**C12**	0.87 ± 0.19	1.07 ± 0.22	3.84 ± 1.46	1.14 ± 0.30
**C14:2**	0.63 ± 0.13	2.35 ± 0.61	2.06 ± 0.63	2.38 ± 0.81
**C14:1**	1.67 ± 0.40	2.30 ± 0.48	8.44 ± 3.30	2.53 ± 0.75
**C14**	2.54 ± 0.58	2.32 ± 0.38	9.35 ± 3.60	2.77 ± 0.52
**C16:2**	0.85 ± 0.20	1.92 ± 0.36	1.75 ± 0.54	2.01 ± 0.43^b^
**C16:1**	5.18 ± 1.30	3.55 ± 0.30	10.65 ± 3.43	4.10 ± 0.68
**C16**	14.40 ± 3.05	9.78 ± 1.27	28.43 ± 9.44	11.28 ± 1.53
**C18:2**	12.62 ± 2.58	15.57 ± 1.49	16.10 ± 3.55	19.56 ± 3.24
**C18:1**	31.96 ± 7.33	19.77 ± 1.81	47.24 ± 10.62	27.89 ± 4.38
**C18**	4.75 ± 0.87	3.88 ± 0.54	10.27 ± 2.88	4.73 ± 0.72
**C20:2**	0.11 ± 0.02	0.09 ± 0.01	0.20 ± 0.08	0.12 ± 0.01
**C20:1**	0.20 ± 0.03	0.15 ± 0.02	0.43 ± 0.14	0.19 ± 0.03
**C20**	0.06 ± 0.01	0.14 ± 0.02^a^	0.18 ± 0.06	0.15 ± 0.03^a^
**Short-acylcarnitines**	16.04 ± 3.43	29.38 ± 4.48^b^	28.88 ± 5.69	29.73 ± 5.52^b^
**Medium-acylcarnitine**	3.17 ± 0.60	6.83 ± 1.47	22.07 ± 7.70	6.38 ± 1.78
**Long-acylcarnitines**	74.97 ± 15.53	61.71 ± 6.19	135.09 ± 36.20	77.59 ± 11.32

Data are expressed as mean ± SEM.

^a^ significantly different from CON after.

^b^ tending to be different from CON after.

^c^ significantly different from LIPID+CAR before.

## Discussion

In the present study, we aimed to investigate whether free carnitine availability could alleviate lipid-induced insulin resistance. We hypothesized that intravenous infusion of L-carnitine would increase the availability of free carnitine in skeletal muscle, which could prevent the development of lipid-induced insulin resistance and metabolic inflexibility during acute lipid infusion.

In the current study, the intravenous administration of L-carnitine increased plasma free carnitine concentrations to 183 μmol/L. These values exceed normal reference values (22.3–54.8 μmol/L) indicating a state of hypercarnitinemia in the plasma and thus increased plasma free carnitine availability. This level of plasma hypercarnitinemia is comparable to earlier studies that also used L-carnitine infusions of similar dosage to reach hypercarnitinemia in the plasma [21, 35].

Although plasma hypercarnitinemia occurred, no differences in skeletal muscle free carnitine concentration were found upon L-carnitine infusion. This is surprising, as the infusion of insulin has been shown to stimulate uptake of carnitine and combinations of carnitine and insulin have been shown to increase carnitine content in muscle [36]. The uptake of carnitine into the skeletal muscle cells is regulated via the sodium dependent organic cation transporter 2 (OCTN2) and tightly regulated by the sarcolemmal Na^+^/K^+^ ATPase pump activity [36–40]. Inhibition of the Na^+^/K^+^ ATPase pump activity has been shown to decrease carnitine uptake in isolated skeletal muscle cells, illustrating the importance of the Na^+^/K^+^ ATPase pump activity in this sodium dependent uptake of carnitine [39, 41]. Insulin is known to facilitate carnitine uptake into skeletal muscle by increasing the activity of the sarcolemmal Na^+^/K^+^ ATPase pump [36, 38, 42]. Therefore, L-carnitine infusion together with hyperinsulinemia (40mU/m^2^/min) was expected to increase skeletal muscle free carnitine levels, however, this was not the case in the present study. It is yet unclear why carnitine concentrations did not increase in muscle tissue. Although this very much exceeds the scope of the current research, a possible theoretical explanation for the lack of increase in skeletal muscle free carnitine and acetylcarnitine in the current study could be that due to the development of lipid-induced insulin resistance, the expected insulin facilitated increase in Na^+^/K^+^ ATPase pump activity may have been blunted, thereby not leading to enhanced sodium mediated co-transport of carnitine into the skeletal muscle [38, 39, 43–45]. However, this remains speculation and future studies will have to investigate potential mechanisms. Unfortunately, we did not perform a clamp with intravenous infusion of L-carnitine, without additional lipid infusion. The latter could have revealed whether lipid infusion indeed hampered carnitine uptake versus insulin infusion alone. Furthermore, the participants of the current study were young and healthy and it is expected that their carnitine availability in muscle was already high to start with. It is conceivable that therefore an increase in muscle carnitine concentration upon infusion is less likely, although this requires further study.

The increase in lipid availability as a result of lipid infusion lead to strongly elevated plasma free fatty acid levels, as reported before [3, 34]. It was previously reported that due to this rise in FFA levels, glucose infusion rates (GIR), insulin sensitivity and metabolic flexibility decreases after 2–4 hours of lipid infusion [3, 4, 34, 46–48]. Indeed, we found that glucose infusion rate and M-value both decreased by approximately 50% indicating a marked induction of insulin resistance upon lipid infusion. Furthermore, carbohydrate oxidation was reduced and lipid oxidation increased in the insulin-stimulated state, reflecting a blunted metabolic flexibility upon insulin stimulation. However, these changes were similar in the conditions with or without infusion of L-carnitine. As carnitine needs to be taken up in the muscle to exert an effect on insulin sensitivity according to our hypothesis, it is not be surprising that insulin sensitivity was not affected in the present study. In contrast to our findings, beneficial effects of L-carnitine infusion has been reported previously in overweight patients with type 2 diabetes. Thus, in these studies, intravenous infusion of L-carnitine during a hyperinsulinemic-euglycemic clamp (40mU/kg/min) was shown to improve whole-body glucose disposal [20, 21]. Furthermore, Mingrone et al. [20] reported enhanced insulin stimulated glucose oxidation upon L-carnitine infusion during a clamp (40mU/kg/min), reflecting improved metabolic flexibility. However, in these studies, skeletal muscle free carnitine availability is not reported. It should be noted that in these studies, no lipid infusion was used and therefore, no lipid-induced insulin resistance occurred and the uptake of carnitine may have been more efficiently stimulated by insulin. Whether improved skeletal muscle free carnitine availability indeed underlies the beneficial metabolic effects that were reported previously, remains to be shown.

In the current study, plasma acetylcarnitine concentrations were reduced upon insulin stimulation in the control trial. Next to acetylcarnitine levels, reduced short-, medium- and long-chain acylcarnitine levels have been reported in situations of hyperinsulinemia. We here confirmed this reduction in short-, medium- and long-chain acylcarnitines levels upon insulin infusion. These decreases in acylcarnitine species are likely to reflect a decreased lipid oxidation caused by hyperinsulinemia, as previously reported [49, 50]. Indeed, decreased lipid oxidation and increased glucose oxidation were observed upon hyperinsulinemia in the control trial. Lipid infusion increased plasma acetylcarnitine, medium- and long-chain acylcarnitines, probably reflecting increased efflux of β-oxidation intermediates by tissues such as liver and muscle [51, 52]. The main contributor to the plasma acetylcarnitine elevations might be increased production by β-oxidation and subsequently release of acetylcarnitine by the liver, as indicated by earlier studies using a porcine animal model or human volunteers to assess trans-organ acylcarnitine fluxes [52, 53]. Plasma C3 acylcarnitines and the sum of plasma short-chain acylcarnitines (C3 to C5) did not change upon lipid infusion, contrary to the other acylcarnitine species. Since C3 is mainly derived from branched-chain amino acids, this might explain the different kinetics. With additional intravenous carnitine infusion (LIPID+CAR), an even more pronounced increase in plasma acetylcarnitine, medium- and long-chain acylcarnitines compared to only lipid infusion, was observed. Similarly, C3 and short-chain acylcarnitine were also increased upon combined lipid + carnitine infusion compared to lipid infusion alone (p<0.05). As plasma acylcarnitine concentrations are significantly higher upon carnitine infusion, these data indicate the necessity of free carnitine availability in the formation of acylcarnitine species suggests that carnitine infusion can further stimulate the efflux of β-oxidation intermediates from the liver.

Surprisingly, skeletal muscle acetylcarnitine concentrations remained unaffected by lipid infusion as well as lipid combined with L-carnitine infusion. Contrary, Tsintzas et al. [48] reported increased skeletal muscle acetylcarnitine concentrations upon lipid infusion. Although we cannot provide a direct explanation for this discrepancy, the more than two-fold higher plasma FFA concentration in the study of Tsintzas might be of relevance. Future research is necessary to unravel what is underlying this difference.

Furthermore, skeletal muscle short-chain acylcarnitine (C3-C5) levels decreased upon insulin infusion in the control trial. Medium- and long-chain acylcarnitine seemed to decrease as well, although not reaching significance. Insulin reduces lipolysis resulting in decreased plasma FFA availability, and as a consequence, glucose oxidation increases. The decrease in skeletal muscle acylcarnitine species upon insulin therefore probably reflects this decreased FFA availability resulting in a transition of lipid towards glucose oxidation induced by hyperinsulinemia [49, 50]. Lipid infusion increased plasma FFA concentrations despite high insulin concentration. Therefore, the decrease in short- and medium-chain acylcarnitines in skeletal muscle tissue as found in the control trial upon insulin was blunted upon the combination of lipid and insulin infusion, which may indicate higher skeletal muscle lipid oxidation rates upon the elevation of plasma lipids by lipid infusion. Remarkably, this effect was only seen on the short and medium chain acylcarnitine species and not on the long chain species: lipid infusion did not blunt the insulin-induced reduction in long-chain acylcarnitine species. Although we cannot provide a direct explanation for this effect, it could be speculated that during acute lipid overload, accumulation of β-oxidation intermediates does mainly happen at later passages through the β-oxidation.

A study limitation is the low number of participants in the current study. Although the number of participants is quite low (n = 8), a cross-over design was used to bolster the power of this mechanistic study. According to our sample size calculation, 13 participants needed to perform the entire study. Upon eight finalized participants, an interim analysis was performed. We calculated that based on the difference found in insulin sensitivity in the current study (eight participants), over five hundred participants would have been needed to render the effect of carnitine supplementation on insulin sensitivity statistically significant. Considering the reproducibility of the methods used, the sample size of n = 8 should be sufficient to be able to pick up a change in insulin sensitivity of 10%, which is assumed to be clinically relevant. Thus, it can be inferred from the observed results that if there is a difference between treatments, it is so small that it is not clinically relevant. Therefore, the study was ended prematurely after eight participants. A second limitation of the current study is the fact that we only included men. The hormonal changes that occur in women as result of the menstrual cycle are known to affect insulin sensitivity [54, 55] and would have had to be taken into account. This would have made the execution of the current study with a three arm cross-over design very complex.

In summary and conclusion, lipid infusion strongly increased plasma FFA levels and resulted in a hampered metabolic flexibility and insulin sensitivity. The addition of intravenous infusion of L-carnitine elevated plasma free carnitine availability as expected. However, against expectations, L-carnitine infusion did not increase skeletal muscle free carnitine availability, possibly due to insulin resistance of the OCTN2 receptor involved in skeletal muscle carnitine uptake. Since skeletal muscle free carnitine availability remained unaltered with L-carnitine infusion, we cannot conclude on the original hypothesis whether free carnitine availability in skeletal muscle tissue might be crucial in maintaining metabolic flexibility and insulin sensitivity. Therefore, further research is necessary to unravel if skeletal muscle free carnitine availability is indeed crucial in maintaining metabolic flexibility and insulin sensitivity. Using an acute carnitine treatment, the current study was performed to mechanistically investigate the role of L-carnitine. However, to investigate whether carnitine treatment would be beneficial in improving insulin sensitivity in clinical practice, future studies using long-term carnitine treatment needs to be performed.

## Supporting information

S1 Checklist CONSORT 2010 checklist of information to include when reporting a randomised trial*. (DOC)

S1 File (PDF)

## References

[1] Kelley DE, Mandarino LJ. Fuel selection in human skeletal muscle in insulin resistance: a reexamination. Diabetes. 2000;49(5):677–83. Epub 2000/07/25. 10.2337/diabetes.49.5.677 . 10905472

[2] Perseghin G, Ghosh S, Gerow K, Shulman GI. Metabolic defects in lean nondiabetic offspring of NIDDM parents: a cross-sectional study. Diabetes. 1997;46(6):1001–9. Epub 1997/06/01. 10.2337/diab.46.6.1001 . 9166672

[3] Meex RC, Phielix E, Moonen-Kornips E, Schrauwen P, Hesselink MK. Stimulation of human whole-body energy expenditure by salsalate is fueled by higher lipid oxidation under fasting conditions and by higher oxidative glucose disposal under insulin-stimulated conditions. The Journal of clinical endocrinology and metabolism. 2011;96(5):1415–23. Epub 2011/02/04. 10.1210/jc.2010-1816 . 21289240

[4] Phielix E, Meex R, Ouwens DM, Sparks L, Hoeks J, Schaart G, et al High oxidative capacity due to chronic exercise training attenuates lipid-induced insulin resistance. Diabetes. 2012;61(10):2472–8. Epub 2012/07/13. 10.2337/db11-1832 . 22787138 PMC3447923

[5] Goodpaster BH, He J, Watkins S, Kelley DE. Skeletal muscle lipid content and insulin resistance: evidence for a paradox in endurance-trained athletes. The Journal of clinical endocrinology and metabolism. 2001;86(12):5755–61. Epub 2001/12/12. 10.1210/jcem.86.12.8075 . 11739435

[6] Muoio DM, Noland RC, Kovalik JP, Seiler SE, Davies MN, DeBalsi KL, et al Muscle-specific deletion of carnitine acetyltransferase compromises glucose tolerance and metabolic flexibility. Cell metabolism. 2012;15(5):764–77. Epub 2012/05/09. 10.1016/j.cmet.2012.04.005 . 22560225 PMC3348515

[7] Noland RC, Koves TR, Seiler SE, Lum H, Lust RM, Ilkayeva O, et al Carnitine insufficiency caused by aging and overnutrition compromises mitochondrial performance and metabolic control. The Journal of biological chemistry. 2009;284(34):22840–52. Epub 2009/06/26. 10.1074/jbc.M109.032888 . 19553674 PMC2755692

[8] Stephens FB, Constantin-Teodosiu D, Greenhaff PL. New insights concerning the role of carnitine in the regulation of fuel metabolism in skeletal muscle. The Journal of physiology. 2007;581(Pt 2):431–44. Epub 2007/03/03. 10.1113/jphysiol.2006.125799 . 17331998 PMC2075186

[9] Bieber LL. Carnitine. Annual review of biochemistry. 1988;57:261–83. Epub 1988/01/01. 10.1146/annurev.bi.57.070188.001401 . 3052273

[10] McGarry JD, Sen A, Esser V, Woeltje KF, Weis B, Foster DW. New insights into the mitochondrial carnitine palmitoyltransferase enzyme system. Biochimie. 1991;73(1):77–84. Epub 1991/01/01. 10.1016/0300-9084(91)90078-f . 2031961

[11] Power RA, Hulver MW, Zhang JY, Dubois J, Marchand RM, Ilkayeva O, et al Carnitine revisited: potential use as adjunctive treatment in diabetes. Diabetologia. 2007;50(4):824–32. Epub 2007/02/21. 10.1007/s00125-007-0605-4 . 17310372 PMC5682624

[12] Schooneman MG, Houtkooper RH, Hollak CE, Wanders RJ, Vaz FM, Soeters MR, et al The impact of altered carnitine availability on acylcarnitine metabolism, energy expenditure and glucose tolerance in diet-induced obese mice. Biochimica et biophysica acta. 2016;1862(8):1375–82. Epub 2016/04/27. 10.1016/j.bbadis.2016.04.012 . 27112275

[13] Wessels B, van den Broek NM, Ciapaite J, Houten SM, Wanders RJ, Nicolay K, et al Carnitine supplementation in high-fat diet-fed rats does not ameliorate lipid-induced skeletal muscle mitochondrial dysfunction in vivo. American journal of physiology Endocrinology and metabolism. 2015;309(7):E670–8. Epub 2015/08/20. 10.1152/ajpendo.00144.2015 . 26286868

[14] Lindeboom L, Nabuurs CI, Hoeks J, Brouwers B, Phielix E, Kooi ME, et al Long-echo time MR spectroscopy for skeletal muscle acetylcarnitine detection. The Journal of clinical investigation. 2014;124(11):4915–25. Epub 2014/10/02. 10.1172/JCI74830 . 25271624 PMC4347229

[15] Ringseis R, Keller J, Eder K. Role of carnitine in the regulation of glucose homeostasis and insulin sensitivity: evidence from in vivo and in vitro studies with carnitine supplementation and carnitine deficiency. European journal of nutrition. 2012;51(1):1–18. Epub 2011/12/03. 10.1007/s00394-011-0284-2 . 22134503

[16] Malaguarnera M, Gargante MP, Russo C, Antic T, Vacante M, Malaguarnera M, et al L-carnitine supplementation to diet: a new tool in treatment of nonalcoholic steatohepatitis—a randomized and controlled clinical trial. The American journal of gastroenterology. 2010;105(6):1338–45. Epub 2010/01/14. 10.1038/ajg.2009.719 . 20068559

[17] Rahbar AR, Shakerhosseini R, Saadat N, Taleban F, Pordal A, Gollestan B. Effect of L-carnitine on plasma glycemic and lipidemic profile in patients with type II diabetes mellitus. European journal of clinical nutrition. 2005;59(4):592–6. Epub 2005/03/03. 10.1038/sj.ejcn.1602109 . 15741989

[18] Molfino A, Cascino A, Conte C, Ramaccini C, Rossi Fanelli F, Laviano A. Caloric restriction and L-carnitine administration improves insulin sensitivity in patients with impaired glucose metabolism. JPEN Journal of parenteral and enteral nutrition. 2010;34(3):295–9. Epub 2010/05/15. 10.1177/0148607109353440 . 20467011

[19] Capaldo B, Napoli R, Di Bonito P, Albano G, Sacca L. Carnitine improves peripheral glucose disposal in non-insulin-dependent diabetic patients. Diabetes research and clinical practice. 1991;14(3):191–5. Epub 1991/12/01. 10.1016/0168-8227(91)90020-e . 1778112

[20] Mingrone G, Greco AV, Capristo E, Benedetti G, Giancaterini A, De Gaetano A, et al L-carnitine improves glucose disposal in type 2 diabetic patients. Journal of the American College of Nutrition. 1999;18(1):77–82. Epub 1999/03/06. 10.1080/07315724.1999.10718830 . 10067662

[21] Giancaterini A, De Gaetano A, Mingrone G, Gniuli D, Liverani E, Capristo E, et al Acetyl-L-carnitine infusion increases glucose disposal in type 2 diabetic patients. Metabolism: clinical and experimental. 2000;49(6):704–8. Epub 2000/07/06. 10.1053/meta.2000.6250 . 10877193

[22] Snedecor GWa C W.G. Statistical Methods. Ames: Iowa State University Press; 1980.

[23] Dempster P, Aitkens S. A new air displacement method for the determination of human body composition. Medicine and science in sports and exercise. 1995;27(12):1692–7. Epub 1995/12/01. . 8614327

[24] Vink RG, Roumans NJ, Arkenbosch LA, Mariman EC, van Baak MA. The effect of rate of weight loss on long-term weight regain in adults with overweight and obesity. Obesity (Silver Spring, Md). 2016;24(2):321–7. Epub 2016/01/28. 10.1002/oby.21346 . 26813524

[25] Siri WE. Body composition from fluid spaces and density: analysis of methods. 1961. Nutrition (Burbank, Los Angeles County, Calif). 1993;9(5):480–91; discussion, 92. Epub 1993/09/01. . 8286893

[26] Bilet L, van de Weijer T, Hesselink MK, Glatz JF, Lamb HJ, Wildberger J, et al Exercise-induced modulation of cardiac lipid content in healthy lean young men. Basic research in cardiology. 2011;106(2):307–15. Epub 2010/12/25. 10.1007/s00395-010-0144-x . 21181177 PMC3032894

[27] Peronnet F, Massicotte D. Table of nonprotein respiratory quotient: an update. Canadian journal of sport sciences = Journal canadien des sciences du sport. 1991;16(1):23–9. Epub 1991/03/01. . 1645211

[28] Bergstrom J, Hermansen L, Hultman E, Saltin B. Diet, muscle glycogen and physical performance. Acta physiologica Scandinavica. 1967;71(2):140–50. Epub 1967/10/01. 10.1111/j.1748-1716.1967.tb03720.x . 5584523

[29] van Vlies N, Tian L, Overmars H, Bootsma AH, Kulik W, Wanders RJ, et al Characterization of carnitine and fatty acid metabolism in the long-chain acyl-CoA dehydrogenase-deficient mouse. The Biochemical journal. 2005;387(Pt 1):185–93. Epub 2004/11/13. 10.1042/BJ20041489 . 15535801 PMC1134946

[30] Vreken P, van Lint AE, Bootsma AH, Overmars H, Wanders RJ, van Gennip AH. Quantitative plasma acylcarnitine analysis using electrospray tandem mass spectrometry for the diagnosis of organic acidaemias and fatty acid oxidation defects. Journal of inherited metabolic disease. 1999;22(3):302–6. Epub 1999/06/29. 10.1023/a:1005587617745 . 10384392

[31] DeFronzo RA, Tobin JD, Andres R. Glucose clamp technique: a method for quantifying insulin secretion and resistance. The American journal of physiology. 1979;237(3):E214–23. Epub 1979/09/01. 10.1152/ajpendo.1979.237.3.E214 . 382871

[32] Le DS, Brookshire T, Krakoff J, Bunt JC. Repeatability and reproducibility of the hyperinsulinemic-euglycemic clamp and the tracer dilution technique in a controlled inpatient setting. Metabolism: clinical and experimental. 2009;58(3):304–10. Epub 2009/02/17. 10.1016/j.metabol.2008.09.029 . 19217443 PMC2692526

[33] Thomsen C, Storm H, Christiansen C, Rasmussen OW, Larsen MK, Hermansen K. The day-to-day variation in insulin sensitivity in non-insulin-dependent diabetes mellitus patients assessed by the hyperinsulinemic-euglycemic clamp method. Metabolism: clinical and experimental. 1997;46(4):374–6. Epub 1997/04/01. 10.1016/s0026-0495(97)90050-0 . 9109838

[34] Hoeks J, Mensink M, Hesselink MK, Ekroos K, Schrauwen P. Long- and medium-chain fatty acids induce insulin resistance to a similar extent in humans despite marked differences in muscle fat accumulation. The Journal of clinical endocrinology and metabolism. 2012;97(1):208–16. Epub 2011/10/28. 10.1210/jc.2011-1884 . 22031508

[35] Natali A, Santoro D, Brandi LS, Faraggiana D, Ciociaro D, Pecori N, et al Effects of acute hypercarnitinemia during increased fatty substrate oxidation in man. Metabolism: clinical and experimental. 1993;42(5):594–600. Epub 1993/05/01. 10.1016/0026-0495(93)90218-d . 8492714

[36] Stephens FB, Constantin-Teodosiu D, Laithwaite D, Simpson EJ, Greenhaff PL. Insulin stimulates L-carnitine accumulation in human skeletal muscle. FASEB journal: official publication of the Federation of American Societies for Experimental Biology. 2006;20(2):377–9. Epub 2005/12/22. 10.1096/fj.05-4985fje . 16368715

[37] Clausen T. Na+-K+ pump regulation and skeletal muscle contractility. Physiological reviews. 2003;83(4):1269–324. Epub 2003/09/25. 10.1152/physrev.00011.2003 . 14506306

[38] Deachapunya C, Palmer-Densmore M, O’Grady SM. Insulin stimulates transepithelial sodium transport by activation of a protein phosphatase that increases Na-K ATPase activity in endometrial epithelial cells. The Journal of general physiology. 1999;114(4):561–74. Epub 1999/09/25. 10.1085/jgp.114.4.561 . 10498674 PMC2229463

[39] Rebouche CJ. Carnitine movement across muscle cell membranes. Studies in isolated rat muscle. Biochimica et biophysica acta. 1977;471(1):145–55. Epub 1977/11/15. 10.1016/0005-2736(77)90402-3 . 921970

[40] Tamai I, Ohashi R, Nezu J, Yabuuchi H, Oku A, Shimane M, et al Molecular and functional identification of sodium ion-dependent, high affinity human carnitine transporter OCTN2. The Journal of biological chemistry. 1998;273(32):20378–82. Epub 1998/08/01. 10.1074/jbc.273.32.20378 . 9685390

[41] Georges B, Le Borgne F, Galland S, Isoir M, Ecosse D, Grand-Jean F, et al Carnitine transport into muscular cells. Inhibition of transport and cell growth by mildronate. Biochemical pharmacology. 2000;59(11):1357–63. Epub 2001/02/07. 10.1016/s0006-2952(00)00265-3 . 10751544

[42] Pochini L, Scalise M, Galluccio M, Indiveri C. OCTN cation transporters in health and disease: role as drug targets and assay development. Journal of biomolecular screening. 2013;18(8):851–67. Epub 2013/06/19. 10.1177/1087057113493006 . 23771822

[43] Record RD, Froelich LL, Vlahos CJ, Blazer-Yost BL. Phosphatidylinositol 3-kinase activation is required for insulin-stimulated sodium transport in A6 cells. The American journal of physiology. 1998;274(4 Pt 1):E611–7. Epub 1998/05/12. 10.1152/ajpendo.1998.274.4.E611 . 9575821

[44] Rodriguez-Commes J, Isales C, Kalghati L, Gasalla-Herraiz J, Hayslett JP. Mechanism of insulin-stimulated electrogenic sodium transport. Kidney international. 1994;46(3):666–74. Epub 1994/09/01. 10.1038/ki.1994.319 . 7996787

[45] Ragolia L, Cherpalis B, Srinivasan M, Begum N. Role of serine/threonine protein phosphatases in insulin regulation of Na+/K+-ATPase activity in cultured rat skeletal muscle cells. The Journal of biological chemistry. 1997;272(38):23653–8. Epub 1997/09/20. 10.1074/jbc.272.38.23653 . 9295306

[46] Boden G, Jadali F, White J, Liang Y, Mozzoli M, Chen X, et al Effects of fat on insulin-stimulated carbohydrate metabolism in normal men. The Journal of clinical investigation. 1991;88(3):960–6. Epub 1991/09/01. 10.1172/JCI115399 . 1885781 PMC295496

[47] Hoeks J, Hesselink MK, Russell AP, Mensink M, Saris WH, Mensink RP, et al Peroxisome proliferator-activated receptor-gamma coactivator-1 and insulin resistance: acute effect of fatty acids. Diabetologia. 2006;49(10):2419–26. Epub 2006/08/10. 10.1007/s00125-006-0369-2 . 16896940

[48] Tsintzas K, Chokkalingam K, Jewell K, Norton L, Macdonald IA, Constantin-Teodosiu D. Elevated free fatty acids attenuate the insulin-induced suppression of PDK4 gene expression in human skeletal muscle: potential role of intramuscular long-chain acyl-coenzyme A. The Journal of clinical endocrinology and metabolism. 2007;92(10):3967–72. Epub 2007/07/27. 10.1210/jc.2007-1104 . 17652214

[49] Consitt LA, Koves TR, Muoio DM, Nakazawa M, Newton CA, Houmard JA. Plasma acylcarnitines during insulin stimulation in humans are reflective of age-related metabolic dysfunction. Biochemical and biophysical research communications. 2016;479(4):868–74. Epub 2016/10/04. 10.1016/j.bbrc.2016.09.116 . 27693789 PMC5067238

[50] Soeters MR, Sauerwein HP, Duran M, Wanders RJ, Ackermans MT, Fliers E, et al Muscle acylcarnitines during short-term fasting in lean healthy men. Clinical science (London, England: 1979). 2009;116(7):585–92. Epub 2008/10/24. 10.1042/cs20080433 . 18945215

[51] Koves TR, Ussher JR, Noland RC, Slentz D, Mosedale M, Ilkayeva O, et al Mitochondrial overload and incomplete fatty acid oxidation contribute to skeletal muscle insulin resistance. Cell metabolism. 2008;7(1):45–56. Epub 2008/01/08. 10.1016/j.cmet.2007.10.013 . 18177724

[52] Xu G, Hansen JS, Zhao XJ, Chen S, Hoene M, Wang XL, et al Liver and Muscle Contribute Differently to the Plasma Acylcarnitine Pool During Fasting and Exercise in Humans. The Journal of clinical endocrinology and metabolism. 2016;101(12):5044–52. Epub 2016/09/21. 10.1210/jc.2016-1859 . 27648961

[53] Schooneman MG, Ten Have GA, van Vlies N, Houten SM, Deutz NE, Soeters MR. Transorgan fluxes in a porcine model reveal a central role for liver in acylcarnitine metabolism. American journal of physiology Endocrinology and metabolism. 2015;309(3):E256–64. Epub 2015/06/04. 10.1152/ajpendo.00503.2014 . 26037250

[54] Yeung EH, Zhang C, Mumford SL, Ye A, Trevisan M, Chen L, et al Longitudinal study of insulin resistance and sex hormones over the menstrual cycle: the BioCycle Study. The Journal of clinical endocrinology and metabolism. 2010;95(12):5435–42. Epub 2010/09/17. 10.1210/jc.2010-0702 . 20843950 PMC2999972

[55] Livingstone C, Collison M. Sex steroids and insulin resistance. Clinical science (London, England: 1979). 2002;102(2):151–66. Epub 2002/02/09. 10.1042/cs1020151 . 11834135

