# Supplementary material for: L-carnitine infusion does not alleviate lipid-induced insulin resistance and metabolic inflexibility
Source: PLoS One. 2020 Sep 25;15(9):e0239506. doi: 10.1371/journal.pone.0239506 (PMC7518598; doi:10.1371/journal.pone.0239506)
Supplement: S1 File — (PDF) [file pone.0239506.s002.pdf]

Impact of L-Carnitine infusion on Lipid induced Insulin resistance

(Version 5: November 2016)

PROTOCOL TITLE : Impact of L-Carnitine infusion on Lipid induced Insulin resistance

**Protocol ID**	
**Short title**	**Carnitine infusion and Insulin resistance**
**EudraCT number**	**2016-000810-31**
**Version**	**5**
**Date**	**November 2016**
**Coordinating investigator/project leader**	**Dr. V. Schrauwen-Hinderling**<br>***Department of Radiology & Human Biology***<br>***Maastricht University***<br>***PO Box 616, 6200 MD Maastricht***<br>**E-mail: v.schrauwen@maastrichtuniversity.nl**
**Principal investigator(s) (in Dutch: hoofdonderzoeker/ uitvoerder)**<br>***<Multicenter research: per site>***	**Drs. Yvonne Bruls**<br>***Department of Radiology***<br>***Maastricht University Hospital***<br>***PO Box 5800, 6202 AZ Maastricht***<br>**E-mail: y.bruls@maastrichtuniversity.nl**<br><br>**Dr. V. Schrauwen-Hinderling**<br>***Department of Radiology & Human Biology***<br>***Maastricht University***<br>***PO Box 616, 6200 MD Maastricht***<br>**E-mail: v.schrauwen@maastrichtuniversity.nl**
**Sponsor (in Dutch: verrichter/opdrachtgever)**	**Maastricht University**<br>**Nutrition and Toxicology Research Institute Maastricht (NUTRM)**<br>**P.O. Box 616, 6200 MD Maastricht**
**Subsidising party**	**Not applicable**
**Independent expert (s)**	**Ronald Henry**<br>**Afd Interne Geneeskunde**<br>**Postbus 5800 6202AZ Maastricht**<br>**E-mail: rma.henry@mumc.nl**
**Laboratory sites**	**Laboratory of Human Biology (UM) and Haematologie (AZM)**

PROTOCOL SIGNATURE SHEET

Name	Signature	Date
**Head of Department:**<br>**Prof. Jochum Plat**<br>**Universiteitssingel 50**<br>**6229 ER Maastricht**<br>**j.plat@maastrichtuniversity.nl**		
**Principal Investigator:**<br>**Vera Schrauwen-Hinderling**<br>**Universiteitssingel 50**<br>**6229 ER Maastricht**<br>**v.schrauwen@maastrichtuniversity.nl**		

TABLE OF CONTENTS

1. INTRODUCTION AND RATIONALE 10
2. OBJECTIVES 14
3. STUDY DESIGN 15
4. STUDY POPULATION 18
4.1 Population (base) 18
4.2 Inclusion criteria 18
4.3 Exclusion criteria 19
4.4 Sample size calculation 19
5. TREATMENT OF SUBJECTS 20
5.1 Investigational product/treatment 20
5.2 Use of co-intervention (if applicable) 20
5.3 Escape medication (if applicable) 21
6. INVESTIGATIONAL PRODUCT 22
6.1 Name and description of investigational product(s) 22
6.2 Summary of findings from non-clinical studies 22
6.3 Summary of findings from clinical studies 22
6.4 Summary of known and potential risks and benefits 22
6.5 Description and justification of route of administration and dosage 22
6.6 Dosages, dosage modifications and method of administration 23
6.7 Preparation and labelling of Investigational Medicinal Product 23
6.8 Drug accountability 24
7. NON-INVESTIGATIONAL PRODUCT 25
7.1 Name and description of non-investigational product(s) 25
7.2 Summary of findings from non-clinical studies 25
7.3 Summary of findings from clinical studies 25
7.4 Summary of known and potential risks and benefits 25
7.5 Description and justification of route of administration and dosage 25
7.6 Dosages, dosage modifications and method of administration 25
7.7 Preparation and labelling of Non Investigational Medicinal Product 26
7.8 Drug accountability 26
8. METHODS 27
8.1 Study parameters/endpoints 27
8.1.1 Main study parameter/endpoint 27
8.1.2 Secondary study parameters/endpoints (if applicable) 27
8.1.3 Other study parameters (if applicable) 27
8.2 Randomisation, blinding and treatment allocation 27
8.3 Study procedures 28
8.4 Withdrawal of individual subjects 33
8.4.1 Specific criteria for withdrawal (if applicable) 33
8.5 Replacement of individual subjects after withdrawal 33
8.6 Follow-up of subjects withdrawn from treatment 34

8.7 Premature termination of the study....34
9. SAFETY REPORTING....34
9.1 Temporary halt for reasons of subject safety....34
9.2 AEs, SAEs and SUSARs....34
9.2.1 Adverse events (AEs)....34
9.2.2 Serious adverse events (SAEs)....34
9.2.3 Suspected unexpected serious adverse reactions (SUSARs)....35
9.3 Annual safety report....36
9.4 Follow-up of adverse events....36
9.5 [Data Safety Monitoring Board (DSMB) / Safety Committee]....36
10. STATISTICAL ANALYSIS....37
10.1 Primary study parameter(s)....37
10.2 Secondary study parameter(s)....37
10.3 Other study parameters....38
10.4 Interim analysis (if applicable)....38
11. ETHICAL CONSIDERATIONS....39
11.1 Regulation statement....39
11.2 Recruitment and consent....39
11.3 Objection by minors or incapacitated subjects (if applicable)....39
11.4 Benefits and risks assessment, group relatedness....39
11.5 Compensation for injury....41
11.6 Incentives (if applicable)....42
12. ADMINISTRATIVE ASPECTS, MONITORING AND PUBLICATION....43
12.1 Handling and storage of data and documents....43
12.2 Monitoring and Quality Assurance....43
12.3 Amendments....43
12.4 Annual progress report....44
12.5 Temporary halt and (prematurely) end of study report....44
12.6 Public disclosure and publication policy....44
13. STRUCTURED RISK ANALYSIS....45
13.1 Potential issues of concern....45
13.2 Synthesis....46
14. REFERENCES....47

LIST OF ABBREVIATIONS AND RELEVANT DEFINITIONS

**1H-MRS**	**Proton magnetic resonance spectroscopy**
**ABR**	**ABR form, General Assessment and Registration form, is the application form that is required for submission to the accredited Ethics Committee (In Dutch, ABR = Algemene Beoordeling en Registratie)**
**AE**	**Adverse Event**
**AR**	**Adverse Reaction**
**BMI**	**Body mass index**
**CA**	**Competent Authority**
**CCMO**	**Central Committee on Research Involving Human Subjects; in Dutch: Centrale Commissie Mensgebonden Onderzoek**
**CrAT**	**Carnitine Acyl Transferase**
**CV**	**Curriculum Vitae**
**DEXA**	**Dual Energy X-ray Absorptiometry**
**DSMB**	**Data Safety Monitoring Board**
**ECG**	**Electrocardiogram**
**EU**	**European Union**
**EudraCT**	**European drug regulatory affairs Clinical Trials**
**GCP**	**Good Clinical Practice**
**GIR**	**Glucose infusion rate**
**IB**	**Investigator's Brochure**
**IC**	**Informed Consent**
**IMP**	**Investigational Medicinal Product**
**IMPD**	**Investigational Medicinal Product Dossier**
**LIPID+CAR**	**Lipid infusion combined with L-carnitine**
**LIPID+PLAC**	**Lipid infusion combined with placebo as in saline**
**METC**	**Medical research ethics committee (MREC); in Dutch: medisch ethische toetsing commissie (METC)**
**MRI**	**Magnetic resonance imaging**
**MRS**	**Magnetic resonance spectroscopy**
**PDH**	**Pyruvate dehydrogenase**
**Rd**	**Rate of disappearance**
**RER**	**Resting expiratory ratio**
**(S)AE**	**(Serious) Adverse Event**
**SPC**	**Summary of Product Characteristics (in Dutch: officiële**

	productinfomatie IB1-tekst)
Sponsor	The sponsor is the party that commissions the organisation or performance of the research, for example a pharmaceutical company, academic hospital, scientific organisation or investigator. A party that provides funding for a study but does not commission it is not regarded as the sponsor, but referred to as a subsidising party.
SUSAR	Suspected Unexpected Serious Adverse Reaction
T2DM	Type 2 diabetes Mellitus
VO2max	Maximal oxygen uptake
Wbp	Personal Data Protection Act (in Dutch: Wet Bescherming Persoonsgevens)
WMO	Medical Research Involving Human Subjects Act (in Dutch: Wet Medisch-wetenschappelijk Onderzoek met Mensen
Wmax	Maximal wattage

SUMMARY

Rationale: Insulin resistant subjects and type 2 diabetic patients are characterized by a decreased metabolic flexibility: a reduced capability to switch from fat oxidation in the basal state to carbohydrate oxidation in the insulin-stimulated state. This metabolic inflexibility is an early hallmark in the development of diabetes. Recent evidence suggests that a low carnitine availability may limit acetylcarnitine formation, thereby reducing metabolic flexibility. Thus, when substrate flux in the muscle is high, acetyl-CoA concentrations increase, leading to inhibition of pyruvate dehydrogenase (PDH) and thereby reducing glucose oxidation. The conversion of acetyl-CoA to acetylcarnitine relieves this acetyl-CoA pressure on PDH. To provide more direct insight into the effect of carnitine in preventing metabolic inflexibility and insulin resistance and to further explore the mechanism of action is the focus of this research. Here, we hypothesize that the capacity to form acetylcarnitine may rescue lipid-induced insulin resistance. To this end, insulin resistance will be induced by lipid infusion in healthy volunteers and it will be tested whether carnitine co-infusion can alleviate insulin resistance.

Objective: The primary objectives are to investigate whether L-carnitine infusion may rescue lipid-induced insulin resistance and whether L-carnitine infusion is improving metabolic flexibility in the state of lipid-induced insulin resistance. Furthermore, a secondary objective is to examine the molecular pathways of carnitine and acetylcarnitine, responsible for muscle insulin sensitivity.

Study design: The current study is an interventional randomized crossover trial in which each subject serves as it owns control. Subjects will be blinded for the intervention.

Study population: n=13, healthy young (18-40 years) male subjects will be included.

Intervention (if applicable): Thirteen healthy subject will be subjected to the intervention of L-carnitine infusion. To investigate whether L-Carnitine infusion may rescue lipid induced insulin resistance and improve metabolic flexibility three intervention trials are included. The first trial includes lipid infusion combined with L-Carnitine infusion (=LIPID + CAR). In the second trial, L-carnitine infusion will be replaced by placebo infusion in the form of saline (= LIPID + PLAC) in order to investigate the effect of L-Carnitine. During the third trial, lipid infusion will be replaced by infusion of saline and will serve as a control for the lipid infusion (=SALINE + PLAC) and is necessary to investigate to what extend L-carnitine can rescue lipid induced insulin resistance. All three trials will be separated by at least two weeks. Subjects will be blinded, so no information about the infused substances will be provided to them. The three different trials will be allocated in a random order.

Main study parameters/endpoints: The primary study endpoint is whole body insulin sensitivity, measured by the hyperinsulinemic-euglycemic clamp. Secondary endpoints are

maximal acetylcarnitine concentrations after exercise, metabolic compounds in the blood and measurements regarding skeletal muscle metabolism in skeletal muscle tissue obtained by needle biopsies.

Nature and extent of the burden and risks associated with participation, benefit and group relatedness: Subjects will first visit the University once for screening purposes during which length, weight and blood pressure will be measured. An ECG will be performed, blood will be drawn and they will fill in 2 questionnaires. If screening was successfully completed, subjects will visit the university in the morning (fasted) for a Bod Pod measurement (body composition measurement) and a maximal cycling test (visit 2: 1 hour). During the third visit subject will come fasted (for 5 hours, from lunch onwards) to the university in the afternoon (5PM) for a 30 minute cycling test with a magnetic resonance spectroscopy (MRS) measurement immediately before and after cycling to determine exercise-induced acetylcarnitine concentration. Subsequently, subjects visit the University for the three intervention trials (LIPID+CAR, LIPID+PLAC, SALINE+PLAC). These three days will consist of undergoing a hyperinsulinemic euglycemic clamp combined with either lipid, carnitine or saline (11 hours). Furthermore, at the beginning and end of the clamps, a muscle biopsy will be obtained. For these visits, subjects have to report to the university in the morning in the fasted state. Three days in advance, subjects fill out a food diary to monitor their food intake. The evenings prior to these three test days, subjects have to eat a standardize meal (macaroni bolognaise). Muscle biopsies lead to mild discomfort and there is a risk of hematoma. During the hyperinsulinemic euglycemic clamp, a risk of hypoglycaemia exists. In summary, we will draw approximately 538 ml blood during the entire study period. During each of the three intervention trial we draw a maximum of 176 ml blood.

1. INTRODUCTION AND RATIONALE

During the past decades, the prevalence of type 2 diabetes mellitus (T2DM) has increased rapidly worldwide. T2DM is associated with an increased risk of cardiovascular disease and a markedly reduced life expectancy. Consequently, T2DM poses a major burden on our health care system. The recent increase in T2DM prevalence is strongly associated with behavioral risk factors such as overweight and obesity and a lifestyle characterized by physical inactivity. Type 2 diabetic patients and pre-diabetic subjects are characterized by skeletal muscle insulin resistance, identified as reduced insulin-stimulated glucose uptake and oxidation [1]. A diminished metabolic flexibility underlies this insulin resistance of the muscle: The switch from fat oxidation (in the fasted state) to high carbohydrate oxidation (in the postprandial or insulin-stimulated state) and vice versa is markedly reduced compared to healthy subjects. This phenomenon is occurring very early in the pathogenesis of type 2 diabetes. Even in healthy subjects with an increased risk of developing diabetes later in life (offspring of type 2 diabetic patients), metabolic inflexibility is already apparent [2]. Metabolic inflexibility leads to delayed postprandial glucose clearance and thereby to disturbances in glucose homeostasis. Metabolic flexibility is determined by the change in respiratory quotient during a hyperinsulinemic euglycemic clamp, when switching from basal state to insulin-stimulated state. Recently, rodent studies associated insulin resistance and metabolic inflexibility with changes in carnitine status [3], [4]. It was shown that processes like aging and the consumption of a high-fat diet, both well known to lead to insulin resistance and metabolic inflexibility, decreased the availability of free carnitine in mice [3]. The finding that supplementation with carnitine in these models was able to completely restore insulin sensitivity suggests a causal role for carnitine deficiency in the development of insulin resistance [3]. Also in humans, carnitine supplementation as well as carnitine infusion was shown to improve insulin sensitivity and glucose tolerance in insulin resistant subjects with low carnitine status [5], although the mechanism remains elusive. Acute carnitine infusion increases carnitine availability in the skeletal muscle during a hyperinsulinemic euglycemic clamp [6]. Furthermore, carnitine infusion increased insulin sensitivity in type 2 diabetic subjects [7]

Carnitine is best known for its function of enabling fatty acid import into the mitochondria for subsequent oxidation. However, a novel hypothesis – based on recent data – suggests that carnitine also has an important role in the exchange of acetyl groups with acetyl-CoA [8] and therefore functions as a buffer to keep acetyl-CoA concentrations low (carnitine + acetyl-CoA + → acetyl-carnitine + ~CoA, see figure 1). This is very important, as accumulation of acetyl-CoA will lead to the inhibition of pyruvate dehydrogenase (PDH), thereby inhibiting glucose oxidation. **Accumulation of acetyl-CoA levels especially occurs when myocellular substrate influx is very high, such as during exercise, high-fat feeding, but also in subjects characterized by high circulating glucose and fatty acid levels (such as type 2 diabetes).**

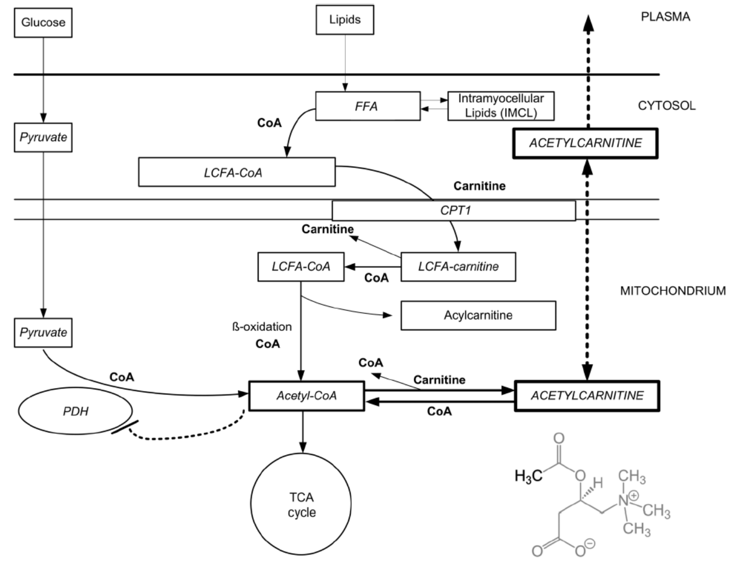

Figure 1:
Schematic overview of the conversion of acetyl-CoA to acetylcarnitine by the enzyme carnitine-acyltransferase (CrAT in order to export acetyl-units from mitochondria (from ref 10)

Therefore, adequate conversion of acetyl-CoA to acetylcarnitine prevents inhibition of PDH activity and therefore allows sustained oxidation of glucose in the post-prandial phase. This rescue reaction is facilitated by the mitochondrial enzyme carnitine acyl transferase (CrAT) in the presence of ample free carnitine.

In animal studies, it was shown that obesity and the consumption of a high-fat diet decreased CrAT activity and diminished the acetylcarnitine/AcetylCoA ratio [9]. Interestingly, muscle-specific knockout of CrAT lead to insulin resistance and metabolic inflexibility in mice with concomitantly decreased PDH activity (see figure 2 from reference [9]. In line with this, gain of CrAT function in human myotubes results in stimulated acetylcarnitine efflux and increased PDH activity (see figure 3 from reference [9]).

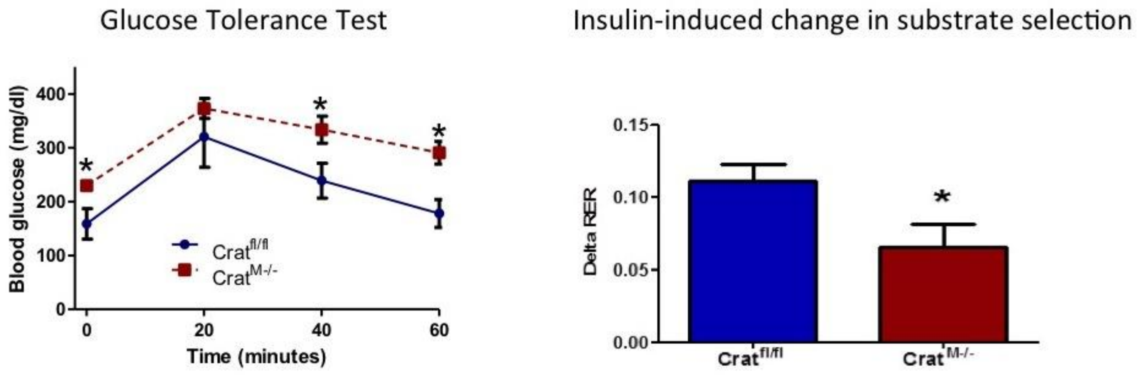

Figure 2: Results from Crat knock out mice (Crat-/-) and control mice (crat fl/fl) showing decreased glucose tolerance and decreased metabolic flexibility in knock out animals where conversion to acetylcarnitine is not possible. From reference [9].

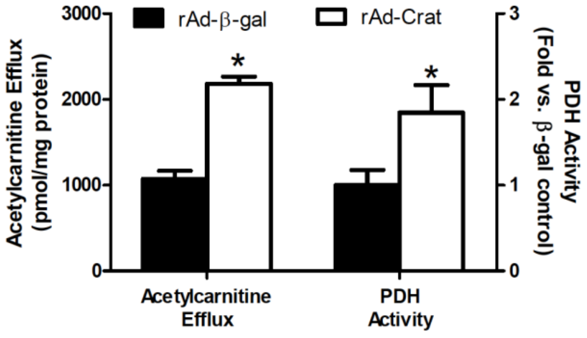

Figure 3: Results from human myotubes, either subjected to gain of Crat function (rAd-□-gal) or loss of function (crAd-Crat) showing increased acetylcarnitine efflux and higher PDH activity with gain of function (from reference 9).

In the case of high substrate availability and low availability of free carnitine (or low CRAT activity), the low conversion of acetyl-CoA to acetylcarnitine is detectable using muscle biopsies or by measuring acetylcarnitine dynamically by MRS. The low carnitine availability - or low CrAT activity - underlying the low acetylcarnitine formation is expected to hamper the switch from fat to glucose oxidation, thereby contributing to metabolic inflexibility. It was shown that, using long echo time ^{1}H-MRS, acetylcarnitine concentrations are also lowered in type 2 diabetic patients and insulin resistant humans (figure 4). We also showed that CrAT activity (determined in vastus lateralis muscle samples) correlates tightly with insulin sensitivity, therefore, also in humans, a low capacity to form acetylcarnitine is characteristic for insulin resistant states.

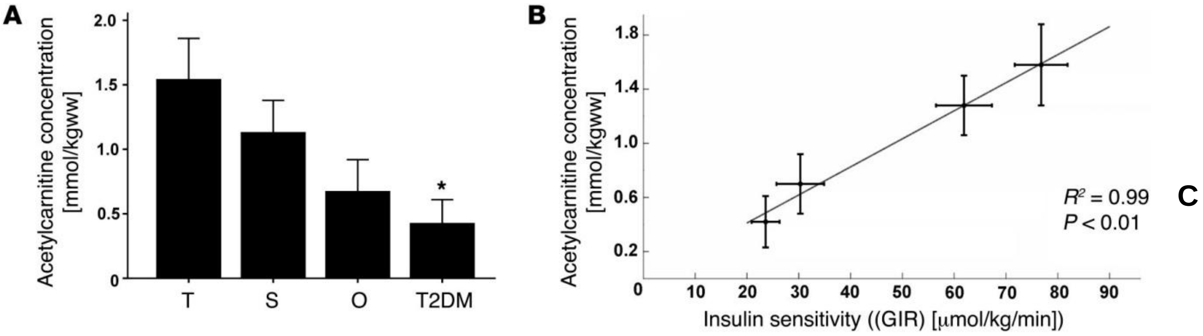

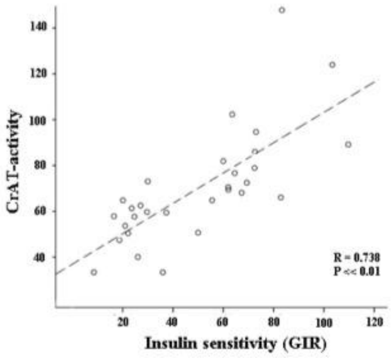

Figure 4: Acetylcarnitine concentrations as determined by long echo time 1H-MRS in four groups of subjects, covering a large spectrum of metabolic healthy (from highly trained young athletes (T), to sedentary young lean controls (S), obese controls (O) and type 2 diabetic patients (T2DM) (panel A). The two first groups (T and S) and the two last groups (O and T2DM) were BMI- and age matched). In these groups, acetylcarnitine concentrations are positively correlated with insulin sensitivity (panel B) and CrAT activity is also correlated with insulin sensitivity (panel C) [10].

In summary, the results from animal studies and preliminary human studies show that carnitine availability and acetylcarnitine concentrations are low in insulin resistant states. Carnitine infusion is able to increase carnitine availability in skeletal muscle and improve insulin sensitivity. However, the mechanisms of action remain unknown. To provide more

direct insight into the effect of carnitine in preventing metabolic inflexibility and insulin resistance and to further explore the mechanism of action we set up this carnitine infusion study. Here, it is hypothesized that the capacity to form acetylcarnitine may rescue lipid-induced insulin resistance, as it was previously shown in animal studies that high fat availability inhibits CrAT activity, which can be compensated by high carnitine concentrations [7]. To this end, insulin resistance will be induced by lipid infusion in healthy volunteers and it will be tested whether carnitine co-infusion can alleviate insulin sensitivity.

2. OBJECTIVES

Primary Objective:

- To investigate whether L-carnitine infusion may rescue lipid-induced insulin resistance
- To investigate whether L-carnitine infusion is improving metabolic flexibility in the state of lipid-induced insulin resistance.

Secondary Objective:

- To examine the molecular pathways of carnitine and acetylcarnitine responsible for muscle insulin sensitivity

3. STUDY DESIGN

In this study we will investigate whole body insulin sensitivity of healthy adults during a 6-hour hyperinsulinemic euglycemic clamp in a placebo controlled randomized cross over design. All subjects will undergo three hyperinsulinemic euglycemic clamps. Two hyperinsulinemic euglycemic clamps will be performed with simultaneous infusion op lipids. In one of these lipid infusion trials, subjects will simultaneously receive L-carnitine infusion (=LIPID + CAR). In the other trial, L-carnitine infusion will be replaced by placebo infusion in the form of saline (=LIPID + PLAC). In a third hyperinsulinemic euglycemic clamp, lipid infusion will be replaced by the infusion of saline and will serve as a control for the lipid infusion (=SALINE + PLAC). All three trials will be separated by at least two weeks. Subjects will be blinded, so no information about the infused substances will be provided to them.

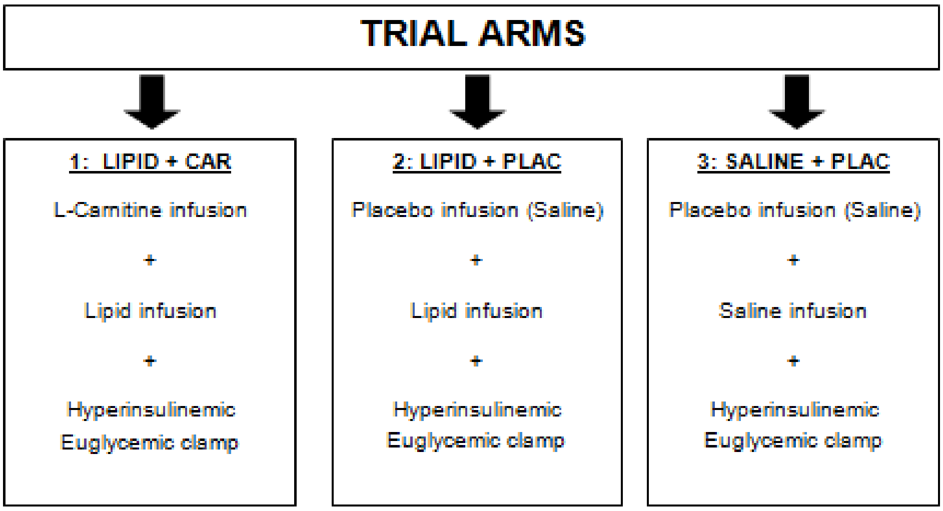

Inclusion: Subjects will be recruited in and near the vicinity of Maastricht by means of posters and advertisements in local newspapers. When subjects react on the advertisement, they will be contacted by telephone. During this telephonic interview the study protocol will be verbally explained to the subject and some of the basic inclusion criteria will be checked, such as weight, height, BMI, age, sports participation, general health, dietary habits, MRI contra-indications and being vegetarian. When this is in compliance with our study protocol, subjects will receive the detailed subject information via email or mail. They will be instructed to read it carefully, and to ask questions when things are unclear. Subjects get at least one week to carefully read all information provided to them. If they still want to participate after carefully reading the subject information and completely understand it, subjects will be invited to the university for signing the informed consent and to be screened subsequently.

Visit 1 (Screening): Subjects will be screened to assess eligibility. On the day of the screening, subjects are invited to the university in the morning after an overnight fast (10PM). Before subjects are screened they have to sign an informed consent form. Subsequently, measurements of height, weight and blood pressure will be performed. Furthermore, an ECG will be administrated and a medical history questionnaire, MRI questionnaire and questions about sports and dietary habits will be filled out by the subjects (Appendix F1.1 and F1.2). Only if all results from the screening visit are in compliance with our inclusion criteria, subjects can participate in the study.

Visit 2 (Bod Pod + VO_2max): Subjects included after the screening visit will enter the study program. For the first visit (visit 1), subjects are invited to the university in the morning after an overnight fast (10PM). During the first study visit, body composition will be accurately determined by Bod Pod. In this way subjects can be more closely characterized. Besides body composition, subjects' maximal oxygen uptake (VO_2max) will be measured during an incremental cycling test in order to determine maximal aerobic capacity. All subjects will be continuously monitored via an ECG device during the entire cycling test for safety reasons. Because subjects need to stay fasted for the Bod Pod measurement, they are asked to take something to eat with them to consume before performing the maximal cycling test.
Visit 3 (^{1}H-MRS cycling measurement): The second visit (visit 2) will include the ^{1}H-MRS cycling measurement. Subjects will come fasted (since noon) to the university at 5PM. An baseline MRI/MRS measurement will be performed to determine acetylcarnitine concentrations in vivo. After this baseline MRI/MRS measurement an 30 minute 70% Wmax (determined during VO_2max visit 1) cycling performance is applied immediately followed by a second MRI/MRS measurement to determine the maximal increase in acetylcarnitine levels caused by exercise (the capacity to form acetylcarnitine).
Visit 4/5/6 (hyperinsulinemic euglycemic clamp): During visit 4,5, and 6 a 6-hour hyperinsulinemic euglycemic clamp measurement will be carried out. During each visit (visit 4,5,6) a different trial arm will be tested (trial arm 1: LIPID + CAR, train arm 2: LIPID + PLAC, trial arm 3: SALINE + PLAC). Subjects will be randomly allocated to the different trials arms. Subjects report to the university after an overnight fast (8PM) at 7AM. We will place an intravenous cannula into the forearm, take a blood sample and begin infusing the glucose tracer (this is part of the preperaring steps for the clamp). Next, we will take the first muscle biopsy. Hereafter, we will continue with a 30 minute indirect calorimetry measurement and the 6-hour hyperinsulinemic euglycemic clamp as described in chapter 8.3. At the end of the clamp we will take a second muscle biopsy of the m. vastus lateralis. The test ends and the subject will receive lunch. The subject will be discharged at 5PM provided blood glucose levels are stable. Three days prior to visit 4,5 and 6, the subjects will be asked to withstand from alcohol, refrain from strenuous physical activity and adhere to their normal eating pattern. During these three days, subjects fill out a food diary to monitor if food intake is equal before the test periods (Appendix F2). Furthermore, subjects will be asked to consume a standardized meal the evening before the 6-hour hyperinsulinemic euglycemic test days (visit 4,5,6), followed by an overnight fast (from 8PM). The meal will be a ready to eat meal "macaroni bolognaise" bought in the local supermarket. Visit 4,5 and 6 will be separated by at least two weeks.

An overview of the entire study is provided in figure 5:

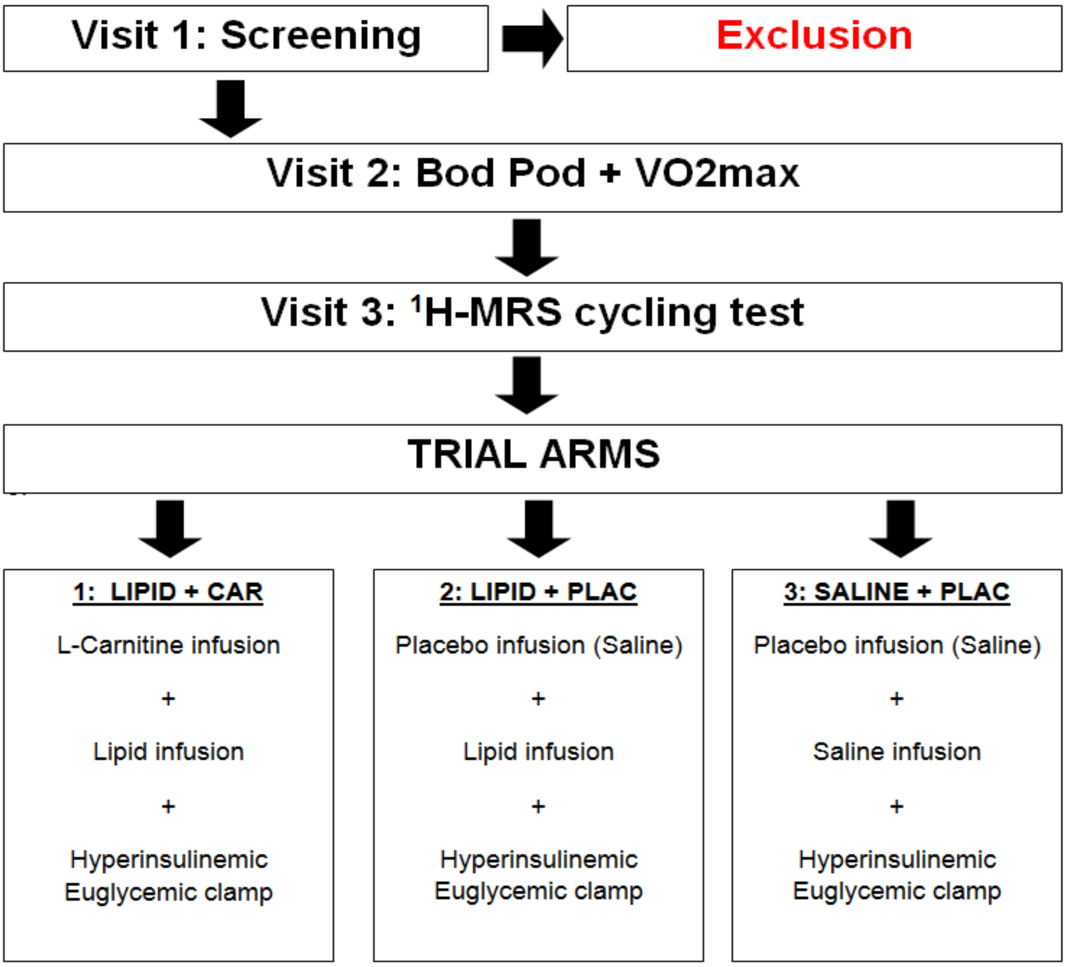

Figure 5: Study overview

The duration of the study is expected to be two years. An overview of all tests performed in the study is illustrated in table 1:

Table 1: Overview of all tests performed

Visit	What	Duration	Notes
1	Screening	1 hour	Overnight fasted (since 10PM)
2	Bod Pod<br>VO_2max	30 minutes<br>30 minutes	Overnight fasted (since 10PM)
3	^{1}H-MRS cycling test	2.5 hours	5 hours fasted (since noon)
4	6-hour hyperinsulinemic euglycemic clamp + 2 muscle biopsies	11 hours	Overnight fasted (since 8PM)
5	6-hour hyperinsulinemic euglycemic clamp + 2 muscle biopsies	11 hours	Overnight fasted (since 8PM)
6	6-hour hyperinsulinemic euglycemic clamp + 2 muscle biopsies	11 hours	Overnight fasted (since 8PM)

4. STUDY POPULATION

4.1 Population (base)

In this study 13 Caucasian healthy lean (BMI: 18-25 kg/m^2) male subjects aged 18-40 years will be tested. The study will be restricted to males to prevent hormonal influences of the menstrual cycle and known gender differences in metabolism. Subjects will be recruited in and near the vicinity of Maastricht by means of posters and advertisements on the university and hospital grounds and in local newspapers (see appendix E2 and E3).

Subjects who are interested and respond to the advertisement will be contacted by telephone. During this telephonic interview the study protocol will be verbally explained to the subjects and some of the basic inclusion and exclusion criteria will be checked such as weight, height, BMI, age, sports participation, general health, dietary habits, MRI contra-indications and being vegetarian. By contacting subjects first on the telephone, we can prevent unnecessary travel burden and time effort for the subjects and study personnel. When the subjects are interested and fulfil the basic inclusion criteria, they will receive the detailed subject-information by e-mail or mail accompanied with a general brochure (provided by the Dutch government) about participating in a medical study. Subjects will be instructed to read it carefully, and ask questions when things are unclear. Permission to contact the subject again after one week of consideration will be asked during the telephonic interview. If subjects still want to participate after carefully reading the subject-information and completely understanding it, they will be invited to the university for signing the informed consent immediately followed by a screening visit. Inviting the subjects to the university provides an extra opportunity for the subjects to ask questions to the researcher personally. The informed consent will be signed and dated by the subjects as well as the researchers in duplicate: one form for the researcher and one form for the subjects. Only when the informed consent is signed, subjects will perform the screening measurements. Subjects have the right to end their co-operation to the study at any time.

4.2 Inclusion criteria

In order to be eligible to participate in this study, a subject must meet all of the following criteria:

- Caucasian
- Healthy (as determined by responsible physician based on a medical questionnaire)
- Male
- Age: 18-40 years
- Normal BMI: 18-25 kg/m^2
- Stable dietary habits
- No use of medication interfering with investigated study parameters (as determined by responsible physician)

4.3 Exclusion criteria

A potential subject who meets any of the following criteria will be excluded from participation in this study:

- Female
- Haemoglobin levels < 7.8 mmol/L
- Uncontrolled hypertension
- Use of anticoagulants
- Engagement in exercise > 3 hours a week
- Being vegetarian or vegan (because of altered whole body carnitine status)
- Smoking
- Alcohol and/or drug abuse
- Unstable body weight (weight gain or loss > 5kg in the last 3 months)
- Significant food allergies/intolerances (seriously hampering study meals)
- Participation in another biomedical study within 1 month before the first study visit, which would possibly hamper our study results
- Medication use known to hamper subject's safety during the study procedures
- Medication use known to interfere with investigated study parameters
- Subjects with contra-indications for MRI
- Subjects who intend to donate blood during the intervention or subjects who have donated blood less than three months before the start of the study
- Subjects who do not want to be informed about unexpected medical findings
- Subjects who do not want that their treating physician is informed

4.4 Sample size calculation

The improvement in insulin sensitivity, determined by a clamp (whole body glucose utilization) combined with carnitine infusion is assumed to be around 0.5 mg/kg/min ± 0.68 mg/kg/min [7, 11-13], therefore μ=0.5 mg/kg/min. The variation in the individual improvement upon carnitine infusion is estimated to be 0.68 mg/kg/min (σ=0.68). Using the following online power calculator for cross-over trials (http://hedwig.mgh.harvard.edu/sample_size/js/js_crossover_quant.html), sample size was calculated. To reach a power (1-β) of 80% and a significance level (α) of 0.05 (one-sided) a minimal sample size of N=13 was calculated according to the online power calculator. One-sided testing is adequate as the hypothesis will be tested that carnitine *improves* insulin sensitivity.

Considering a drop-out of 20% ((13/100)*20 = 3 subjects) we will need to include 3 extra subjects in the study, so a total inclusion of 16 subjects. Taking into account exclusion after screening in 30% of the cases, we will need to screen 21 subjects.

5. TREATMENT OF SUBJECTS

5.1 Investigational product/treatment

This is a cross-over randomized trial, in which each subject will participate in three trial arms (visit 4,5,6). Thus, each subject will act as its own control. In all three trial arms insulin sensitivity will be measured during a 6-hour hyperinsulinemic euglycemic clamp and two muscle biopsies will be obtained. Subjects will begin with either of the trial arms at random order. To investigate if L-carnitine infusion may rescue lipid-induced insulin resistance, we will induce insulin resistance by lipid infusion (arm 1 & 2) and test whether co-infusion of L-carnitine can alleviate insulin sensitivity. Trial arm 1 (LIPID + CAR) is therefore the intervention arm with actual co-infusion of L-carnitine and trial arm 2 will serve as a control arm due to substitution of L-carnitine administration by infusing saline. L-carnitine is therefore the investigational product in this study.

Previous studies investigating carnitine infusion during a hyperinsulinemic euglycemic clamp showed positive effects glucose infusion rate, glucose oxidation and non-oxidative glucose disposal after carnitine infusion compared to saline infusion [7, 17]. These studies infused a total amount of 1 gram carnitine over 3 hours. To achieve similar results in this study, we concluded that per three hours 1 gram of carnitine should be infused. During the current study, carnitine infusion will be six hours combined with lipids. This would result in a total carnitine infusion of approximately 2 grams due to the longer infusion time of carnitine. Natali et al. previously showed that a total amount of 2 grams of carnitine infused combined with lipid infusion results in positive effects on substrate oxidation [20]. Translating these previous studies into infusion rates and taking the maximal allowed doses of carnitine (30mg/kg/day) into account, we will use the following infusion rates. At the onset of L-carnitine infusion a bolus dose of 4mg/kg will be administrated over 10 minutes to rapidly reach a supraphysiological plasma concentration of approximately 200 µmol/L of carnitine. This will be followed by a constant infusion of L-carnitine at 4 mg/kg/h (=0.07mg/kg/min) for the next 350 minutes to maintain hypercarnitinemia and to ensure that plasma carnitine concentration per se will not be rate limiting to muscle carnitine transport [6]. The total amount of intravenously infused L-carnitine will equal 28 mg/kg/day and therefore not exceed the maximal intravenous administration of 30mg/kg/day (Appendix D2).

5.2 Use of co-intervention (if applicable)

Subjects are advised to continue their normal eating pattern during the time of the study, so no change in macronutrient composition of the diet is necessary. Intake of red meat is allowed. Subjects are also advised to maintain their normal living-, activity-, and sleeping pattern. However, 3 days prior to any of the test days, the subjects will be asked to withstand from alcohol and refrain from strenuous physical activity in order to not confound any of the outcome measures. The dietary habits will be registered during the 3 days preceding the clamp with a food diary. Furthermore, they will be asked to consume a standardized meal (“macaroni bolognaise”) the evening before all three hyperinsulinemic euglycemic clamps

(visit 4,5,6) followed by an overnight fast. During the hyperinsulinemic euglycemic clamp subjects will be administrated to a lipid co-infusion in two out of three trial arms

L-carnitine should not be administrated in case of renal insufficiency, therapy with anticoagulants, pregnancy or breast feeding.

5.3 Escape medication (if applicable)

Not applicable.

6. INVESTIGATIONAL PRODUCT

6.1 Name and description of investigational product(s)

Carnitene® (levocarnitine or L-carnitine): solution for infusion

Intralipid: 20%, emulsion for infusion

Natriumchloride: 0,9%, solution for infusion

6.2 Summary of findings from non-clinical studies

Not applicable.

6.3 Summary of findings from clinical studies

For the SPC of L-carnitine (Carnitene®), see appendix D2
For the SPC of Intralipid, see appendix D2
For the SPC of Natriumchloride, see appendix D2

6.4 Summary of known and potential risks and benefits

For the SPC of L-carnitine (Carnitene®), see appendix D2
For the SPC of Intralipid, see appendix D2
For the SPC of Natriumchloride, see appendix D2

6.5 Description and justification of route of administration and dosage

L-Carnitine (Carnitene®) is a medical product for intravenous infusion and will be supplied by a pharmacist of the MUMC pharmacy. L-Carnitine will be provided by Bipharma, Weesp, NL. The L-carnitine for injection will be delivered in a single dose vial with 1g of L-carnitine per 5mL (200mg/mL) and will be packaged with 5 vials per carton. Packaged cartons with 5 vials will be stored at room temperature (15-25˚C).

Intralipid (20%) is a medical product for intravenous infusion and will be supplied by a pharmacist of the MUMC pharmacy. The Intralipid for infusion will be delivered in plastic bags of 250 and/or 500 mL. Bags will be stored at room temperature (15-25˚C).

Natriumchloride (0.9%) is a medical product for intravenous infusion and will be supplied by a pharmacist of the MUMC pharmacy. The Natriumchloride for infusion will be delivered in bags. Bags will be stored at room temperature (15-25˚C).

6.6 Dosages, dosage modifications and method of administration

L-Carnitine will be administrated intravenously as continuous infusion during the 6-hour hyperinsulinemic euglycemic clamp. The administration will start with a bolus of 4mg/kg for 10 minutes. A bolus will be administrated to ensure rapid supraphysiological plasma concentration of approximately 200µmol/L [6]. Subsequently, continuous L-carnitine infusion of 4mg/kg/h (=0.07mg/kg/min) will start for the remaining 350 minutes of the clamp in order to ensure that plasma carnitine concentrations per se will not be rate limiting to muscle carnitine transport [6]. The maximal administered dosage will be 28mg/kg and will not exceed the maximal daily allowed administration dosage of 30mg/kg per day (Appendix D2).

Intralipid will be administrated intravenously as continuous infusion during the 6-hour hyperinsulinemic euglycemic clamp at an infusion rate of 90ml/h. This lipid infusion during a clamp is a technique commonly used in human research to obtain insulin resistance and is commonly performed in our research group. This insulin resistance is defined as a 1.7-2 fold increase in free fatty concentrations. An infusion rate of 90ml/h has been shown previously to result in an almost 2-fold increase in free fatty acids [18,19].
The maximal administered dosage will be 540ml (108 grams of fat). The maximal allowed dosage equals 3 gram fat per kg body weight. For a individual of 70 kg this equals 210 grams of fat, much higher than the administration in this study.

Natriumchloride (0.9%) will be used as control condition for the Intralipid condition. Natriumchloride (0.9%) will be administrated intravenously as continuous infusion during the 6-hour hyperinsulinemic euglycemic clamp at an infusion rate of 90ml/h to make sure the infused volumes in both conditions (LIPID and SALINE) are equal [18,19].

Natriumchloride 0.9% will also be used as control condition for the Carnitine infusion. Natriumchloride 0.9% will be administrated intravenously as continuous infusion during the 6-hour hyperinsulinemic euglycemic clamp. The infusion volume of the natriumchloride will be equal to the infusion volume of carnitine. Since the infusion volume of carnitine is depended on weight this volume will differ per subject. For each individual subject the infusion volume of natriumchloride will be equal to the carnitine infusion volume in ml.

6.7 Preparation and labelling of Investigational Medicinal Product

L-Carnitine will be stored as appropriate in the original packaging of the IMP delivered by the AZM pharmacy. At the moment that there will be a subject, the researcher goes to the AZM pharmacy with a prescription for the L-carnitine. The AZM pharmacy will provide the L-carnitine with an delivery label that meets annex 13. Nothing of the L-carnitine is labelled in stock, so a GMP licence is not required.

Natriumchloride 0.9% (as control condition for Intralipid) will be stored as appropriate in the original packaging of the IMP delivered by the AZM. At the moment that there will be a subject, the researcher goes to the AZM pharmacy with a prescription for the natriumchloride. The AZM pharmacy will provide the natriumchloride with an delivery label

that meets annex 13. Nothing of the natriumchloride is labelled in stock, so a GMP licence is not required.

Intralipid will be stored as appropriate in its original packaging of the IMP delivered by the AZM pharmacy. At the moment that there will be a subject, the researcher goes to the AZM pharmacy with a prescription for the Intralipid. The AZM pharmacy will provide the Intralipid with an delivery label that meets annex 13. Nothing of the Intralipid is labelled in stock, so a GMP licence is not required

6.8 Drug accountability

The L-carnitine is ordered from the MUMC pharmacy, arrives in its original packaging and is stored as appropriate at room temperature (25˚C). We will discard unused portions of an opened vial, as the formulation does not contain a preservative.

Intralipid is ordered from the MUMC pharmacy, arrives in its original packaging and is stored as appropriate.

Natriumchloride 0.9% is ordered from the MUMC pharmacy, arrives in its original packaging and is stored as appropriate.

7. NON-INVESTIGATIONAL PRODUCT

7.1 Name and description of non-investigational product(s)

Lidocaine-hydrochloride: Local anesthetic solution for injection.

Insulin aspart: Peptide hormone, solution for injection.

Glucose intravenous infusion: 20% w/v, solution for injection.

D-glucose (6,6D2) intravenous solution: Stable isotope tracer, solution for injection.

Meals: Standardized meals are chosen to control food intake the evening before all clamp test visits (visit 4, 5 & 6). Meals are bought from a local supermarket and provided to subject by the researcher.

7.2 Summary of findings from non-clinical studies

Not applicable

7.3 Summary of findings from clinical studies

For the SPC of lidocaine, see appendix D2
For the SPC of insulin, see appendix D2
For the SPC of glucose, see appendix D2
For the IMPD of 6,6D2 tracer glucose, see appendix D2

7.4 Summary of known and potential risks and benefits

For the SPC of lidocaine, see appendix D2
For the SPC of insulin, see appendix D2
For the SPC of glucose, see appendix D2
For the IMPD of 6,6D2 tracer glucose, see appendix D2

7.5 Description and justification of route of administration and dosage

Not applicable. As in standard medical practise.

7.6 Dosages, dosage modifications and method of administration

Lidocaine will be administered subcutaneously and under the muscle fascia at the site of the muscle biopsies. The maximum dosage will not be exceeded in our study protocol, despite multiple injections. For more information see appendix K6.

Insulin will be administered intravenously as continuous infusion during the 6-hour hyperinsulinemic euglycemic clamp. The maximum dosage will not exceed 40 $mU/m^2/min$.

Glucose infusion will be administered intravenously at variable infusion rates to achieve euglycemia.

6,6-D2 tracer glucose will be administered intravenously as continuous infusion. Maximum dosage will not exceed 0.04 mg/kg/min.

7.7 Preparation and labelling of Non Investigational Medicinal Product

The Lidocaine will be stored as appropriate and transferred to a syringe right before administration.

Tracer glucose will be stored as appropriate and transferred to a syringe right before administration.

Insulin will be stored as appropriate and prepared right before administration. Upon preparation of the insulin infusion, the syringe containing the 0.5 mL insulin solution will be marked with the concentration and ingredients: 0.5 mL insulin / 47.5 mL NaCl / 2 mL blood.

Glucose 20% (500mL infusion bag, Baxter) will be stored as appropriate and prepared right before administration. Upon the addition of 64.5 mL 6,6-D2 tracer glucose (stable isotope) and 5.0 mL KCl (7,5%), the glucose bag will be labelled as: + 64.5 mL 6,6-D2 tracer glucose / + 5.0 mL KCl.

7.8 Drug accountability

The Lidocaine is ordered from the MUMC pharmacy, arrives in its original packaging and is stored as appropriate.

Insulin and glucose infusion are ordered from the MUMC pharmacy, arrive in original packaging and are stored appropriately.

Tracer glucose is ordered from the UMC pharmacy (Nijmegen, The Netherlands) and arrives in its original packaging.

The study meals are bought at a local supermarket and stored at 4-8˚C as appropriate. These study meals are taken home by the subject. The subject will be responsible for the appropriate storage of these meals.

8. METHODS

8.1 Study parameters/endpoints

8.1.1 Main study parameter/endpoint

6-hour hyperinsulinemic euglycemic clamp combined with tracer kinetics and indirect calorimetry:

- Whole body insulin sensitivity measured as GIR in µmol/kg/min during the stable period of the insulin phase of the clamp.
- Peripheral insulin sensitivity measured as R_d in µmol/kg/min.
- Metabolic flexibility (delta RER between basal and insulin stimulated state).

The hyperinsulinemic euglycemic clamp technique is considered the gold standard for assessing insulin sensitivity and metabolic flexibility [15].

8.1.2 Secondary study parameters/endpoints (if applicable)

-Maximal acetylcarnitine concentrations after exercise (measured using the ^{1}H-MRS cycling measurement)

-Metabolites in the blood before and during insulin stimulation (i.e. glucose, free fatty acids, triglycerides, cholesterol, insulin)

- CrAT activity (measured in muscle biopsies)
- Acylcarntine profiles (measured in muscle biopsies)
- Lipid and lipid intermediates (measured in muscle biopsies)

A insulin stimulated muscle biopsy is taken at the end of the clamp to investigate insulin sensitivity on a molecular level. This will lead to more mechanistic insight in the mechanism by which carnitine exerts its effects.

8.1.3 Other study parameters (if applicable)

- Body composition (measured using Bod Pod), to assess fat mass, lean body mass and total body mass.
- Maximal aerobic capacity (measured during a VO_2max cycling test)

8.2 Randomisation, blinding and treatment allocation

After inclusion in the study, subjects will be randomly allocated to one of the three interventions arms (saline, intra-lipid, intra-lipid + carnitine). Randomisation will be carried out using an online randomization tool. The intervention arms are labelled according to the subsequent numbers: 1=saline, 2=intra-lipid, 3=intra-lipid+carnitine. The randomisation list is attached in appendix 1A, showing the generated list in excel format. “Set 1” will correspond to the first subject, “set 2” to the second subject and so on. Upon inclusion of a new subject, the subject code will be inserted in consecutive order of inclusion in the excel sheet in the cells that are named “set 1”, “set 2” etc.

The leg of the first muscle biopsy of all muscle biopsies will be randomised (left/right). Hereby, potential influence of difference between leg muscle on the outcome will be attenuated. Randomisation will be carried out using an online randomization tool, comparable to the intervention arm randomisation. The legs are labelled according to the subsequent numbers: 1=left leg, 2=right leg. The randomisation list is attached in appendix 1B.

8.3 Study procedures

Screening

Eligibility for participation in the study will be checked via a telephonic interview prior to inviting subjects for a screening visit at the university. During this telephonic interview the study protocol will be verbally explained to the subjects and some of the basic inclusion and exclusion criteria will be checked, such as weight, height, BMI, age, sports participation, general health, dietary habits, MRI contra-indications and being vegetarian. When the subjects are still interested in participation in the study and fulfil the basic inclusion criteria, they will receive the detailed subject-information by e-mail or mail accompanied with a general brochure (provided by the Dutch government) about participating in a medical study. The subject will be instructed to read it carefully, and to ask questions when things are unclear. Permission to contact the subject again after one week of consideration will be asked during the telephonic interview. If subjects are still willing to participate after reading the information, an appointment for singing informed consent and a screening visit will be made. For this screening visit, subjects will come to the university in the morning after an overnight fast (from 10PM). Before the start of the screening, an informed consent form will be signed and dated by the subjects as well as the researchers in duplicate. One form for the researcher and one form for the subject. The screening visit will include:

- A medical history questionnaire (Appendix F1.1)
- A MRI contra-indication questionnaire (Appendix F1.2)
- Answering questions about lifestyle such as eating and sporting habits (subjects lifestyle should be consistent with in- and exclusion criteria).
- Measurement of length, body weight (E1200, August Sauter, GmbH, Albstadt, Germany) and waist circumference
- Blood pressure
- Electrocardiogram (evaluated by a responsible physician or a colleague appointed by him)
- A fasted blood draw (to determine general health with parameters such as hemoglobin and potassium)

The duration of the entire screening visit will be 1 hour. Only if all the results from the screening visit are in compliance with our in- and exclusion criteria, subjects can participate in the study.

Body composition (Bod Pod)

A Bod Pod measurement will be used to determine body composition and will be performed in the morning in the fasted state (fasted for at least 2 hours prior to arrival at the university). Bod Pod assesses total body volume highly accurately and thereby measures body soft tissue composition (muscle mass and fat mass). By measuring the body's muscle mass and fat mass total body fat percentage can be determined. The total duration of this measurement will be approximately 30 minutes. The measurement per se will take 5 minutes and is performed twice. Some additional time is calculated for changing clothes.

Maximal aerobic capacity test (VO_2max cycling test)

Subjects' maximal oxygen uptake (VO_2max) will be measured by a incremental cycling test to determine maximal aerobic capacity. To determine maximal aerobic capacity, VO_2max and Wmax will be measured on an electronically braked cycle ergometer (Lode Excalibur, Groningen, The Netherlands) during an incremental exhaustive exercise test. In order to do so, O_2 consumption and CO_2 production will be measured using an Oxycon beta. In addition, RER will be monitored as a verification of the subjects maximal effort. After a warming-up period of five minutes, on a start workload of 75 watt, the workload will be increased with 50 watt every 2.5 minutes. When the heart rate of subjects reaches 80% of their predicted maximal heart rate (220-age), the workload will be increased with 25 watt until exhaustion is reached or until the subject is no longer able to keep their speed of rotation above 60 rates per minute. The cycling test will be performed under continuous ECG-monitoring as a safety precaution. The total duration of this measurement will be approximately 30 minutes.

1H-MRS cycling measurement

During the 1H-MRS cycling measurement, acetylcarnitine concentrations will be determined in vivo using proton magnetic resonance spectroscopy (1H-MRS) before and immediately after a 30 minute cycling exercise. Subjects are asked to come fasted (since 12AM) to the university in the afternoon (4.30PM) and bring MRI compatible sporting clothes with them. After arrival at the university, the pre-exercise ^{1}H-MRS measurement will be performed (duration ± 45 minutes). Subsequently, subjects start a 30 minute acetylcarnitine formation exercise. This includes 30 minutes cycling at 70% of the subjects maximal wattage. Immediately after the 30-minutes cycling, a post-exercise ^{1}H-MRS measurement is performed to investigate differences in acetylcarnitine formation as a result of exercise (duration ± 45 minutes). The total duration of the MRS cycling measurement (including explanations and changing of clothes) will be 2.5 hours (finish time around 7PM).

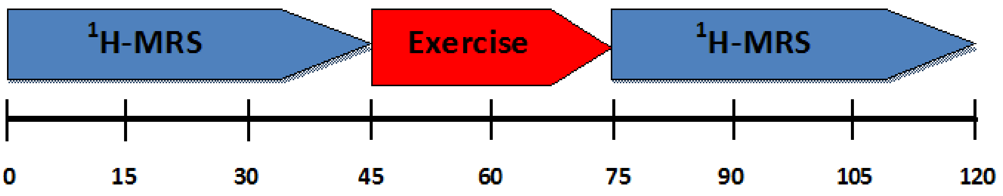

Acetyl-carnitine concentration (by MRI/^{1}H-MRS, duration ca 45 minutes)
Acetyl-carnitine quantification will be performed by proton magnetic resonance spectroscopy (^{1}H-MRS) on a 3.0 T clinical MRI scanner (Achieva, Phillips Healthcare, Best, The Netherlands) of the department of Radiology at the University Hospital Maastricht.
The subject will be installed in the supine position with a surface coil fixed around the upper leg. The subject will be shifted with the legs into the bore of the magnet and is asked to lie still during the time of the measurement. The head of the subject will stay outside the bore of the magnet, making the measurement possible even for moderate claustrophobic subjects. Subjects will receive a buzzer to let the researcher know when they are uncomfortable and they will stay in contact with the researcher via an intercom communication system.
Magnetic resonance imaging (MRI) will be used to guide the spectroscopic measurements and fine shimming will be performed to optimize the magnetic field homogeneity within the region of interest. A volume of interest will be selected within the vastus lateralis from the MRI images and the ^{1}H-MRS spectra will be acquired from this region of interest. The measurement will be repeated with various echo times to determine T2 relaxation kinetics in each subject individually. This is necessary because a sequence with long echo times will be used, giving rise to T2 relaxation, which needs to be corrected for. The intensity of the creatine signal will be used as internal reference.
All the MRS scans will be performed by the same operator, at the same location, and at the same time of the day.

Subjects with a pacemaker, neurostimulator, iron-containing corpora aliena in eyes or brain will be excluded from MRS. Depending on the material, hearing aids and artificial (heart) valves can also be a contraindication. MRS and MRI are modern diagnostic tools that do not imply significant risks (no ionizing radiation). The acquisition of MRI images is accompanied by a 'clanging' noise. Therefore, subjects will be provided with protective headphones. There is a chance that MRI reveals an unexpected medical condition, of which the subject will be informed. His physician will also be informed.

Analysis of ^{1}H-MRS spectra
Spectra will be fitted with the j-MRUI software (www.mrui.uab.es), to determine the intensity of the acetyl-carnitine and creatine peaks (areas under the curve). Both metabolites will be individually corrected for T2 relaxation. To determine individual T2 relaxation time, an exponential curve will be fitted to the signal intensities acquired at different echo times using Matlab (Mathworks Inc). The ratio of the corrected acetyl-carnitine resonance and the corrected creatine resonance will be calculated. Absolute concentrations will be calculated by assuming a constant tissue concentration of creatine of 30 mmol/kg.

Acetylcarnitine formation exercise test
A 30 minutes acetylcarnitine formation exercise test at 70% of the subjects maximal wattage will be performed. The 70% of Wmax is determined during the $VO2_{max}$ cycling test. A protocol of relatively high intensity is chosen to maximize acetylcarnitine production. A fixed

duration and wattage is chosen to prevent variation in exercise intensity and duration between subjects. This cycling protocol is also applied in previous studies with an older research population (40-70 years old overweigthed subjects) and therefore also applicable in the current study with a younger and fitter study population. The 30 minute cycling test will be performed in the room next to the MRI scanner to prevent prolonged time intervals between the end of the cycling measurement and the beginning of the MRS/MRI measurement. No indirect calorimetrie will be performed during this 30 minute acetylcarnitine formation exercise test.

Hyperinsulinemic euglycemic clamp

On the morning of the test day, subjects will report to the university in the fasted state (fasted since 8PM the day before). A teflon cannula will be inserted into an antecubital vein and will be used for the infusion of glucose tracer, glucose, insulin, lipid/saline and carnitine/saline. A ‘manifold’ will be attached to the cannula (see figure 6a below). Separate infusion lines will be connected to this manifold for the infusion of the glucose tracer, glucose, insulin, lipid/saline and carnitine/saline. Blood will be sampled from a second cannula, inserted into a superficial dorsal hand vein. This venous blood will be arterialized by placing the hand into a hot-box, which blows warm air (~65 ºC). After taking a fasting blood sample (7mL), a primed (2.4 mg/kg) constant infusion (0.04 ml/kg/min) of the glucose tracer ([6,6-D2]glucose) is initiated (t=0). This is a naturally occurring isotope, which is in no way harmful to humans and will be obtained from Cambridge Isotope Laboratories, inc. (Andover, MA, USA). Subsequently, a percutaneous muscle biopsy of the vastus lateralis muscle will be taken (see appendix K6 for the SOP muscle biopsy). After 90 minutes infusion of 6,6-D2 glucose, to allow isotopic equilibration, four additional blood samples will be drawn (7ml for each blood sample at t=90,100, 110 and 120). In addition, during the last 30 minutes of this basal period (t=90-120), substrate oxidation is measured with indirect calorimetry (ventilated hood).

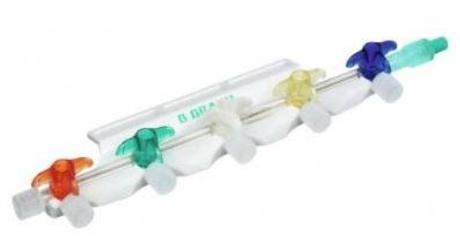

Figure 6A: Manifold for the connection of the separate infusion lines

Insulin sensitivity will be measured by performing simultaneously a 6-hour hyperinsulinemic euglycemic clamp, using the technique of DeFronzo et al. [15]. At t=120, insulin infusion (Novorapid, Novo Nordisk) will be started at a fixed rate (40 mU/m2/min) from 0 to 6 hours (t=120 until t=480). At the same time a variable co-infusion of 20% glucose will be started. At regular time points (every 5 to 10 minutes), a small volume of blood (1.0mL) will be sampled for immediate determination of plasma glucose concentration. When necessary, glucose infusion rate will be adjusted to obtain plasma glucose levels of ~5 mmol/L (euglycemia). After 330 minutes infusion of insulin, four additional blood samples will be drawn (7ml for

each blood sample at t=450, 460, 470 and 480). In addition, during the last 30 minutes of this hyperinsulinemic period (t=450-480), substrate oxidation is measured with indirect calorimetry (ventilated hood). Hereafter, whole body insulin sensitivity can be expressed as the rate of glucose appearance (Ra) and disappearance (Rd).

In two intervention arms (LIPID+PLAC and LIPID+CAR), a lipid emulsion (Intralipid 20%, Fresenius-Kabi) will be administered. This emulsion is based on long-chain fatty acids (10% safflower and 10% soybean oil) and will be administered at a rate of 90ml/h for 6 hours (starting at t=0). In the control trial, saline will be infused. In the LIPID+CAR condition intravenous L-carnitine infusion will take place. At the onset of l-carnitine infusion (t=0) a bolus dose of 4 mg/kg will be administered over 10 min to rapidly reach a supraphysiological plasma concentration of approximately 200 µmol/liter. This will be followed by a constant infusion at 4 mg/kg/h for the remaining of the clamp. In the non-carnitine conditions saline will be infused matching for the total volume infused with carnitine infusion.

Additional blood samples (7mL) will be drawn at t=180, t=240, t=300, t=360 and t=420 for determination of glucose, FFA, insulin and triglycerides. Between t=240 and t=270 an additional ventilated hood measurement will be performed to assess substrate oxidation. At t=480 a muscle biopsy will be taken in all three intervention arms (LIPID+CAR / LIPID+PLAC / SALINE+PLAC). Thereafter insulin 6,6-D2 glucose, lipid/saline and carnitine/saline infusion will be stopped, while glucose infusion will be continued to prevent hypoglycemia, and subjects will be offered a lunch. An overview of the blood draws taken during the clamp is provided in appendix 2A. An overview of the total amount of blood taken during the entire study is calculated in appendix 2B.

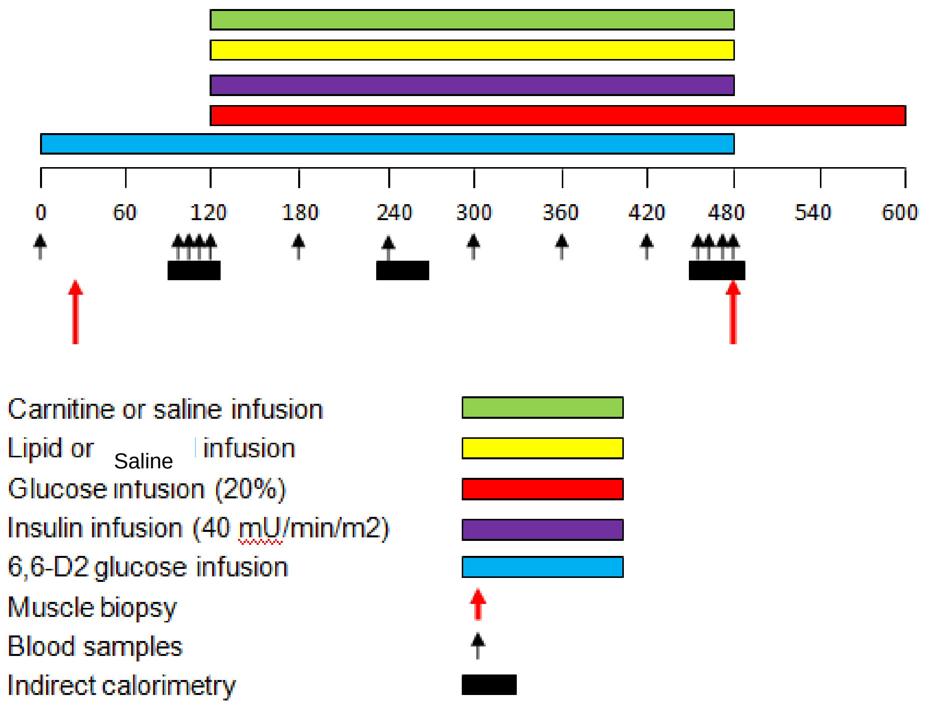

Figure 6b: overview hyperinsulinemic euglycemic clamp

De SOP hyperinsulinemic euglycemic clamp is added in appendix K6. The time of insulin administration (6 hours) deviates from the SOP (2.5 hours) due to the additional infusion of Intralipid in this study. Lipid infusion during a clamp is often performed in the metabolic field and also several times before in our research group via the design explained above. As a result of the additional lipid infusion it does not take 2.5 hours to achieve stable glucose values but 6 hours. These six hours are necessary because it takes 3 to 4 hours until insulin resistance occurs regarding glucose infusion rates. The SOP is followed on all the other aspects.

Muscle biopsy

In total 6 muscle biopsies will be taken during the entire study. Before and at the end of 6-hour hyperinsulinemic euglycemic clamp in all three intervention arms (saline infusion, lipid infusion, lipid infusion + carnitine) a muscle biopsy will be taken. The biopsies will be taken from the Vastus Lateralis muscle via a 5mm diameter side cutting needle after local anesthesia with Lidocaine (1.0% Lidocaine without adrenaline), according to the Bergstrom technique [16] (for a detailed description see the SOP in appendix K6). Biopsies will be taken alternately from both legs, and the first biopsy of each subjects will be randomised. So a random determination of muscle biopsy in the left or right leg will be followed (see section 8.2 and appendix 1B). Muscle tissue will be used for immunohistochemical analysis and for culturing of muscle cells, in order to be able to do full characterization of the phenotype of these muscle cells. The amount of muscle tissue needed for these analysis is about 300 mg; this is the normal size of a muscle biopsy.

8.4 Withdrawal of individual subjects

Subjects can leave the study at any time for any reason if they wish to do so without any consequences. The investigator can decide to withdraw a subject from the study for urgent medical or study related reasons.

8.4.1 Specific criteria for withdrawal (if applicable)

Subjects will be withdraw from the study if the research team becomes aware of any of the following conditions:

- non medical reasons (subjects' request or non compliance with the treatment regimen)
- medical reasons considered significant by the subject and/or investigator
- protocol violation

In case of illness or use of medication the subject and/or the principal investigator with the physician will decide whether or not the subject may continue the study.

8.5 Replacement of individual subjects after withdrawal

Subjects will be replaced after withdrawal

8.6 Follow-up of subjects withdrawn from treatment

After withdrawal, no follow-up of the subject will take place. In case of withdrawal due to medical complications, subjects will be guided to adequate care by the responsible physician if necessary, or subjects will be referred to a general physician.

8.7 Premature termination of the study

Not applicable.

9. SAFETY REPORTING

9.1 Temporary halt for reasons of subject safety

In accordance to section 10, subsection 4, of the WMO, the sponsor will suspend the study if there is sufficient ground that continuation of the study will jeopardise subject health or safety. The sponsor will notify the accredited METC without undue delay of a temporary halt including the reason for such an action. The study will be suspended pending a further positive decision by the accredited METC. The investigator will take care that all subjects are kept informed.

9.2 AEs, SAEs and SUSARs

9.2.1 Adverse events (AEs)

Adverse events are defined as any undesirable experience occurring to a subject during the study, whether or not considered related to L-carnitine (Carnitene®). All adverse events reported spontaneously by the subject or observed by the investigator or his staff will be recorded.

9.2.2 Serious adverse events (SAEs)

A serious adverse event is any untoward medical occurrence or effect that

- results in death;
- is life threatening (at the time of the event);
- requires hospitalisation or prolongation of existing inpatients' hospitalisation;
- results in persistent or significant disability or incapacity;
- is a congenital anomaly or birth defect; or
- any other important medical event that did not result in any of the outcomes listed above due to medical or surgical intervention but could have been based upon appropriate judgement by the investigator.

An elective hospital admission will not be considered as a serious adverse event.

The investigator will report all SAEs to the sponsor without undue delay after obtaining knowledge of the events.

The sponsor will report the SAEs through the web portal *ToetsingOnline* to the accredited METC that approved the protocol, within 7 days of first knowledge for SAEs that result in death or are life threatening followed by a period of maximum of 8 days to complete the initial preliminary report. All other SAEs will be reported within a period of maximum 15 days after the sponsor has first knowledge of the serious adverse events.

9.2.3 Suspected unexpected serious adverse reactions (SUSARs)

Adverse reactions are all untoward and unintended responses to an investigational product related to any dose administered.

Unexpected adverse reactions are SUSARs if the following three conditions are met:

1. the event must be serious (see chapter 9.2.2);
2. there must be a certain degree of probability that the event is a harmful and an undesirable reaction to the medicinal product under investigation, regardless of the administered dose;
3. the adverse reaction must be unexpected, that is to say, the nature and severity of the adverse reaction are not in agreement with the product information as recorded in:
    - Summary of Product Characteristics (SPC) for an authorised medicinal product;
    - Investigator’s Brochure for an unauthorised medicinal product.

The sponsor will report expedited the following SUSARs through the web portal *ToetsingOnline* to the METC:

- SUSARs that have arisen in the clinical trial that was assessed by the METC;
- SUSARs that have arisen in other clinical trials of the same sponsor and with the same medicinal product, and that could have consequences for the safety of the subjects involved in the clinical trial that was assessed by the METC.

The remaining SUSARs are recorded in an overview list (line-listing) that will be submitted once every half year to the METC. This line-listing provides an overview of all SUSARs from the study medicine, accompanied by a brief report highlighting the main points of concern.

The expedited reporting of SUSARs through the web portal Eudravigilance or ToetsingOnline is sufficient as notification to the competent authority.

The sponsor will report expedited all SUSARs to the competent authorities in other Member States, according to the requirements of the Member States.

The expedited reporting will occur not later than 15 days after the sponsor has first knowledge of the adverse reactions. For fatal or life threatening cases the term will be maximal 7 days for a preliminary report with another 8 days for completion of the report.

9.3 Annual safety report

In addition to the expedited reporting of SUSARs, the sponsor will submit, once a year throughout the clinical trial, a safety report to the accredited METC, competent authority, and competent authorities of the concerned Member States.

This safety report consists of:

- a list of all suspected (unexpected or expected) serious adverse reactions, along with an aggregated summary table of all reported serious adverse reactions, ordered by organ system, per study;
- a report concerning the safety of the subjects, consisting of a complete safety analysis and an evaluation of the balance between the efficacy and the harmfulness of the medicine under investigation.

9.4 Follow-up of adverse events

All AEs will be followed until they have abated, or until a stable situation has been reached. Depending on the event, follow up may require additional tests or medical procedures as indicated, and/or referral to the general physician or a medical specialist.

SAEs need to be reported till end of study within the Netherlands, as defined in the protocol

9.5 [Data Safety Monitoring Board (DSMB) / Safety Committee]

Not applicable.

10. STATISTICAL ANALYSIS

For all study parameters data will be reported as minimum, maximum, mean ± SD and/or ± SEM. Statistical analysis will be performed using the statistical computer program SPSS. The Kolmogorov-Smirnov normality test will be performed to evaluate normality distribution. Pearson correlation coefficients will be computed to correlate parameter values. Differences will be considered significant when $p<0.05$.

Missing values will not be replaced. If a missing value occurs for a specific parameter, the subject in question will not contribute to the analysis of that parameter.

10.1 Primary study parameter(s)

Insulin sensitivity
Insulin sensitivity will be measured by means of a 6-hour hyperinsulinemic euglycemic clamp. Insulin sensitivity will be presented quantitatively for:
• Whole body insulin sensitivity (primarily muscle): GIR (µmol/kg/min).
• Peripheral insulin sensitivity: Rd (µmol/kg/min).

Metabolic flexibility
Metabolic flexibility will be computed as the difference in respiratory quotient (RQ) between the basal and insulin stimulate state during the 6-hour hyperinsulinemic euglycemic clamp. The RQ will be computed via $RQ = vCO_2/vO_2$ in which CO_2 and O_2 are measured via the indirect calorimetry.

To find out whether the time course of insulin sensitivity (whole body glucose infusion rate or RD) is different in the three different treatments (LIPID+Carnitine (treatment A), LIPID+Placebo (treatment B) and Saline+Placebo (treatment C)) a two-way ANOVA for repeated measures is used. Therefore, the dependent variable is “insulin sensitivity”, whilst the two factors are the “conditions” (i.e., three groups: “treatment A”: the Lipid+carnitine, “treatment B”: the Lipid+Placebo, “treatment C”: the Saline+Placebo) and “time”. Post-hoc tests will determine where the significant differences are. The metabolic flexibility (Δ RER between basal and insulin stimulated state) will be determined by comparing metabolic flexibility between the conditions with a one-way ANOVA.

10.2 Secondary study parameter(s)

Maximal acetylcarnitine concentrations
Acetylcarnitine concentrations before and after exercise will be presented quantitatively in mmol/kgww. Differences between time-points (pre and post exercise) will be analyzed with a paired sampled T-test. Pearson correlation coefficients will be computed to correlate with parameter values such as insulin sensitivity and metabolic flexibility.

Metabolic compounds

Time effects of metabolites in plasma (i.e. glucose, free fatty acods, triglycerides, cholesterol, insulin) will be presented quantitatively and analyzed using a two way ANOVA repeated measures analysis for analyzing data over time.

Skeletal muscle mRNA and protein levels at baseline and during insulin stimulation
Skeletal muscles parameters of the carnitine mechanism (such as CrAT activity and acetylcarnitine profiles) as well as lipid intermediates will be analyzed comparing pre and post clamp values using a paired sampled T-test. Differences between intervention arms (LIPID+CAR, LIPID+PLAC, SALINE+PLAC) will be analyzed with a one way ANOVA for repeated measured with post-hoc analysis if data allow to do so.

10.3 Other study parameters

Not applicable.

10.4 Interim analysis (if applicable)

An interim analysis will be performed after 3 subjects have completely performed the entire study in order to confirm that the carnitine infusion model upon lipid induced insulin resistance works. It is known that type 2 diabetic patients are characterized by insulin resistance and metabolic inflexibility and that these characteristics are absent in young lean healthy subjects. Infusion of lipids in these young lean healthy subjects results in insulin resistance and decreased metabolic flexibility as in type 2 diabetic patients. This is characterized by a decrease in glucose infusion rate, glucose oxidation and increased fat oxidation rate during the clamp. Carnitine is suggested to rescue metabolic inflexibility and insulin resistance which would result in higher glucose infusion rates, glucose oxidation and decreased fat oxidation upon lipid infusion. To assure that carnitine indeed reaches the muscle we will check whether we see the expected patterns in substrate oxidation with or without addition of carnitine upon lipid infusion, and thus the model used in this study is adequate to answer our research question, we are performing an interim analysis after 3 subjects have completely performed the entire study (all three clamps). During the interim analysis, no new subjects will perform any test days before the results of the interim analysis is known. In case no changes occur in glucose infusion rate or substrate oxidation, the study will not be continued, as in that case, the study set-up is not suited to investigate the molecular mechanisms underlying the beneficial effects of carnitine.

ETHICAL CONSIDERATIONS

10.5 Regulation statement

This study will be conducted according to the principles of the Declaration of Helsinki (64th WMA General Assembly, Fortaleza, Brazil, October 2013) and in accordance with the Medical Research Involving Human Subjects Act (WMO). The study will be conducted in compliance with International Conference on Harmonization Good Clinical Practice. The study will be approved by local medical ethics committee and local authorities before start of the study.

10.6 Recruitment and consent

Subjects will be recruited by means of posters, advertisements in local papers and online (appendix E3) or by contacting subjects from earlier studies, provided that they gave consent for this. Subjects that are interested will be contacted by telephone by a member of the research team. During this telephonic interview the study protocol and the inclusion criteria will be briefly explained to the subjects and some of the basic inclusion criteria will be checked (e.g. gender, age, weight, height, BMI, sports participation, general health, dietary habits, MRI contra-indications and being vegetarian). When the subjects are interested, they will receive the detailed subject information via mail or e-mail or personally. They will be instructed to read it carefully and to ask questions when things are unclear. If desired, subjects can contact an independent physician or their own physician. After about 1 week of consideration, the subject is contacted again if he gave permission for this. Permission to contact the subject again after one week of consideration will be asked during the telephonic interview. If the subject wants to participate after reading the information, they are invited for screening. Before the start of the screening, an informed consent form will be signed and dated by the subjects as well as a member of the research team in duplicate. One form for the researcher and one form for the subject.

10.7 Objection by minors or incapacitated subjects (if applicable)

Not applicable.

10.8 Benefits and risks assessment, group relatedness

This study carries no benefits for the subjects and carries minor risks for the subjects. The major burdens consist of a moderate time commitment and multiple (6) muscle biopsies. During visit 4, visit 5 and visit 6 we will take frequent blood samples with a maximum of 176mL, which can be dangerous in case of anemia. This is why subjects with low hemoglobin levels are excluded from participation to minimize this risk. The total amount of

blood taken in the entire study will maximally be 538 mL divided over minimal one month. Blood samples are taken via vena puncture or an intravenous cannula. There is a risk of pain, minor bleeding, bruising or inflammation of the sites of the cannulas on the arm. State of the art techniques, measures of sterility and trained personnel minimize these risks. During the anesthesia and muscle biopsies of the upper leg, subjects will experience some pressure and sometimes pain of a short duration. Due to the anesthesia, the procedure will be almost always painless. There is a small risk of infection or prolonged bleeding of the muscle biopsy site. Infections or continued bleeding on the other hand are very rare. To minimize these risk, subjects using anti-coagulants are excluded from the study. A hematoma can develop after taking the biopsy. Sometimes, numbness of small skin-area may persist for some time after the biopsy, due to damaging small cutaneous nerves. The skin incision will result in a small scar (~ 5 mm). To promote good wound healing, the incision will be sealed with sterile steri-strips and a waterproof band-aid. After taking the biopsy compression to the biopsy site will be applied manually and with a compression bandage to minimize bleeding. State of the art technique, measures of sterility and trained personnel will minimize the before mentioned burdens and risks. Furthermore, the risks will be lower in healthy young subjects. After the biopsies, subjects will experience some muscle pain for 2-3 days. Hyperinsulinemic euglycemic clamping is a procedure we perform routinely in our laboratory without notable complications. In rare occasions subjects exhibit symptoms of hypoglycemia (even if their blood glucose levels are still above 3 mmol/l). After successfully performing the clamp procedure, blood glucose values will be monitored for an additional 60 minutes with glucose infusion stand-by, in case glucose levels happen to drop. Solid food and sugar-containing drinks will be provided directly after finalizing the clamp to avoid the experience of hypoglycemia.
We do not expect that co-infusion of lipids will be harmful to the subjects in the study because the lipid are administrated in a physiological dose of 540ml (108 gram of lipid) which is far below the maximal allowed dosage of 3 gram fat per kg body weight (For a individual of 70 kg this equals 210 grams of fat, much higher than the administration in this study). There is a small risk of headache, changes in body temperature, tiredness, nausea or vomiting. All side effects are reversible after discontinuation of treatment. In 2006 a comparable lipid infusion study was performed in our research team with healthy young and lean subjects (METC20063049). These subjects received a comparable dosage of Intralipid for 6 hours during a hyperinsulinemic euglycemic clamp, and no complications occurred. This also indicates that the combination of Intralipid with insulin, glucose and tracer glucose is well tolerated.
We do not expect that co-infusion of carnitine will be harmful to the subjects in this study as the human body excretes any surplus via the urine. Carnitine is administered in a dose (28mg/kg body weight/day) which is below the maximal allowed dosage of 30mg/kg body weight/day. There is a small risk of nausea, fishy odor, vomiting or diarrhea. Carnitine infusion is a procedure performed more often in research studies without notable complications [6,7,15,17]. Stephens et al. [6] also performed carnitine infusion during a huperinsulinemic euglycemic clamp for 6 hours. More than double carnitine concentrations (10mg/kg/h) compared to this study were administered to the subjects without complications.

This also indicates that the combination of carnitine with insulin, glucose and tracer glucose is not harmful.
Carnitine in combination with Intralipid is previously investigated by Natali et al. (1993) [20] and no complications were reported. As already noticed before carnitine infusion during a hyperinsulinemic euglycemic clamp as well as Intralipid infusion during a hyperinsulinemic euglycemic clamp were previously performed without noted complications. Therefore, the combination of providing Intralipid and Carnitine together during a hyperinsulinemic euglycemic clamp is not expected to be harmful.
MRS is a safe procedure, with no known health risk as long as none of the exclusion criteria are met. The maximal cycling test and cycling protocol can cause muscle soreness. Measurements performed during the time course of the study can potentially lead to unexpected medical findings. Subjects will be informed about such a finding and possible advised to contact a doctor about this. If a subject does not want to be informed about incidental findings, participation in this study is not possible.
This study will lead to novel insights in the potential beneficial effects of L-carnitine infusion to improve lipid induced insulin resistance and metabolic flexibility. This is expected to have a major impact on our understanding of the muscle metabolism physiology and development and progression of type II diabetes mellitus (T2DM), which will ultimately contribute to new and improved strategies for the prevention, monitoring and therapy of T2DM.

10.9 Compensation for injury

The sponsor/investigator has a liability insurance which is in accordance with article 7 of the WMO.

The sponsor has an insurance which is in accordance with the legal requirements in the Netherlands (Article 7 WMO and the Measure regarding Compulsory Insurance for Clinical Research in Humans of 23th June 2003). This insurance provides cover for damage to research subjects through injury or death caused by the study.

1. € 650.000,-- (i.e. six hundred and fifty thousand Euro) for death or injury for each subject who participates in the Research;
2. € 5.000.000,-- (i.e. five million Euro) for death or injury for all subjects who participate in the Research;
3. € 7.500.000,-- (i.e. seven million and five hundred thousand Euro) for the total damage incurred by the organisation for all damage disclosed by scientific research for the Sponsor as ‘verrichter’ in the meaning of said Act in each year of insurance coverage.

The insurance applies to the damage that becomes apparent during the study or within 4 years after the end of the study.

10.10 Incentives (if applicable)

Subject will be compensated for time investment and the burden of the study, The compensation is based upon the time spent at the university (€8 per our spent during normal work time according to minimum wage) and the amount of muscle biopsies (€15 per biopsy). After complete participation, the total compensation will be €402. Premature termination of the study protocol will result in a reduced compensation relative to the duration of the participation.

Day	Time spent at the university	Financial compensation (Euro)
Visit 1	1 hours	8
Visit 2	1 hours	8
Visit 3	2.5 hours	20
Visit 4	11 hours	88
Visit 5	11 hours	88
Visit 6	11 hours	88
Food diary	1.5 hours (at home)	12
**Total**	**39 hours**	**312**

Subjects will receive an extra financial compensation of €15 for donating one muscle biopsy. Because 2 muscle biopsies are taken (pre and post clamp) at visit 4, visit 5 and visit 6 (in total 6 biopsies) an extra financial compensation of €90 will be provided.
The total financial compensation for the entire study will therefore be €402.

Subjects will not be compensated for the screening only. Subjects will receive compensation for travelling costs, with a maximum of 19 cents per kilometer.

11. ADMINISTRATIVE ASPECTS, MONITORING AND PUBLICATION

11.1 Handling and storage of data and documents

All data will be handled confidentially and coded (concordant with the “wet bescherming persoonsgegevens; WBP”). This means that the study results and human material will be related to a code/number and not to the name, initials or birth-date of the subject. Before the start of the screening, subjects will be assigned a study code that will not change during the study. The code is linked with the name, date of birth and contact-information of the subject in a protected file. Only members of the research team, health inspection (IGZ), METC or monitors can access this file. For all other purposes, the code will be used for subject identification. Furthermore, subjects will be given the opportunity to obtain information regarding their study results. This information will be given verbally or in writing. All blood samples and biopsies taken from the subjects will be stored for 15 years for further analyses in line of this research. Approval will be asked from the subjects on the informed consent form. For future analyses in the line of the current study, approval will be asked again to the METC. The retention period for the research data will be 15 years and only researchers directly involved in the research will have the possibility to access these data. Subjects will be asked in the informed consent form if they may be contacted again for future research.

11.2 Monitoring and Quality Assurance

Not applicable.

11.3 Amendments

A ‘substantial amendment’ is defined as an amendment to the terms of the METC application, or to the protocol or any other supporting documentation, that is likely to affect to a significant degree:

- the safety or physical or mental integrity of the subjects of the trial;
- the scientific value of the trial;
- the conduct or management of the trial; or
- the quality or safety of any intervention used in the trial.

All substantial amendments will be notified to the METC and to the competent authority.

Non-substantial amendments will not be notified to the accredited METC and the competent authority, but will be recorded and filed by the sponsor.

11.4 Annual progress report

The sponsor/investigator will submit a summary of the progress of the trial to the accredited METC once a year. Information will be provided on the date of inclusion of the first subject, numbers of subjects included and numbers of subjects that have completed the trial, serious adverse events/ serious adverse reactions, other problems, and amendments.

11.5 Temporary halt and (prematurely) end of study report

The sponsor will notify the accredited METC and the competent authority of the end of the study within a period of 90 days. The end of the study is defined as the last patient's last visit.

The sponsor will notify the METC immediately of a temporary halt of the study, including the reason of such an action.

In case the study is ended prematurely, the sponsor will notify the accredited METC and the competent authority within 15 days, including the reasons for the premature termination.

Within one year after the end of the study, the investigator/sponsor will submit a final study report with the results of the study, including any publications/abstracts of the study, to the accredited METC and the Competent Authority.

11.6 Public disclosure and publication policy

Publication policy is in agreement with the CCMO publication statement. The results of the study will be published in peer-reviewed scientific journals. Both positive and negative results of the study will be disclosed.

12. STRUCTURED RISK ANALYSIS

12.1 Potential issues of concern

a. Level of knowledge about mechanism of action

The mechanism of the beneficial effects of carnitine are not fully understood, we propose that increased availability of carnitine improves the capacity to form acetyl-carnitine in insulin resistant subjects, thereby increasing the capacity for postprandial glucose oxidation also know as improved metabolic flexibility (see introduction and rational in research protocol).

b. Previous exposure of human beings with the test product(s) and/or products with a similar biological mechanism

L-carnitine for intravenous infusion was used in many clinical studies in similar concentrations than we propose here [6,7,14 and Appendix D2).

c. Can the primary or secondary mechanism be induced in animals and/or in *ex-vivo* human cell material?

Animal research showed indeed that carnitine availability can be limiting in insulin sensitivity and metabolic inflexibility that developed due to high fat exposure (see introduction and rationale) and that addition of carnitine can reverse this. This data is the basis of this research proposal.

d. Selectivity of the mechanism to target tissue in animals and/or human beings

Carnitine is natural compound in skeletal muscle and other tissues of the human body. When consuming a western style diet, availability of free carnitine was shown to decline, especially insulin resistant subject show lower carnitine levels. We hope to reverse this by providing a intravenous L-carnitine infusion in the insulin resistant state (due to lipid infusion).

e. Analysis of potential effect

The highest carnitine dose administered in this study equals 28 mg/kg. This does not exceed the maximal allowed dosage of 30mg/kg/day.

f. Pharmacokinetic considerations

Carnitine is secreted in urine and is usually reabsorbed in the kidneys to a large extent (up to 95% reabsorbtion in case of low carnitine status), leaving room to regulate plasma carnitine levels also by reducing reabsorbtion. L-Carnitine tubular reabsorption exhibits typical characteristics of a carrier-mediated process, including saturation kinetics at high plasma concentrations. The efficiency of the tubular reabsorption of L-carnitine increases as dietary intake decreases in order to maintain circulating L-carnitine concentrations within a narrow normal range. On the other hand, when L-carnitine concentrations increase, such as with exogenous administration via infusion of L-carnitine, greater urinary loss takes places.
This shows that oversupply of carnitine is very unlikely because excess of carnitine can be exreted by the kideneys. As elimination of excess carnitine is depending on a normal kidey function, subjects with plasma concentrations of creatinine higher than 115 micromol/l will be

excluded from the study. There have been no reports of toxicity from l-carnitine overdosage. L-carnitine is easily removed from plasma by dialysis.
The half-life of release of carnitine from skeletal muscle is estimated to be 139 hours.

g. Study population

The study population consists of young (18-40 years) healthy subjects with a normal body weight (BMI:18-25 kg/m^2). . The study is restricted to males to prevent hormonal influences of the menstrual cycle and known gender differences in metabolism. Earlier studies with L-carnitine infusion were performed on the same population ([6] + appendix D2).

h. Interaction with other products

Reports of INR increase with the use of warfarin (anti-coagulant) have been observed. It is recommended that INR levels be monitored in patients on warfarin therapy after the initiation of treatment with l-carnitine or after dose adjustments. To prevent interaction we excluded subjects taking anti-coagulants. Furthermore, no interactions with other products are observed.

i. Predictability of effect

The appearance of side-effects (nausea and diarrhea) cannot be predicted. Transient nausea and vomiting have been observed. Less frequent adverse reactions are body odor, nausea, and gastritis.

j. Can effects be managed?

Effects will vanish when the infusion of the L-carnitine is discontinued.

12.2 Synthesis

L-Carnitine for intravenous infusion is a registered medical product and administration with the proposed dosage is considered safe (SPC L-carnitine: appendix D2) and was used in multiple human studies with similar dosage in similar subject populations.
To limit the appearance of side-effects (nausea and diarrhea), subjects will be screened for renal dysfunction because in these subjects the capacity for homeostatic regulation of carnitine is hampered. The risk of developing side effects in subjects with normal renal function is small and considered acceptable as these are no serious side-effects which can easily be relieved by stopping the L-Carnitine infusion.

13. REFERENCES

1. Kelley, D.E. and L.J. Mandarino, *Fuel selection in human skeletal muscle in insulin resistance: a reexamination.* Diabetes, 2000. 49(5): p. 677-83.
2. Heilbronn, L.K., et al., *Impaired fat oxidation after a single high-fat meal in insulin-sensitive nondiabetic individuals with a family history of type 2 diabetes.* Diabetes, 2007. 56(8): p. 2046-53.
3. Noland, R.C., et al., *Carnitine insufficiency caused by aging and overnutrition compromises mitochondrial performance and metabolic control.* The Journal of biological chemistry, 2009. 284(34): p. 22840-52.
4. Davis M, N.R., Seiler SE, Ikayeva O, Koves TR, Muoio DM. *carnitine acetyltransferase regulates mitochondrial trafficking and metabolic flexibility in skeletal muscle*. in *Keystone symposium: Pathogenesis of Diabetes: Emerging Insights into Molecular Mechanisms*. 2012. Santa Fe, USA.
5. Ringseis, R., J. Keller, and K. Eder, *Role of carnitine in the regulation of glucose homeostasis and insulin sensitivity: evidence from in vivo and in vitro studies with carnitine supplementation and carnitine deficiency.* European journal of nutrition, 2012. 51(1): p. 1-18.
6. Stephens FB, Constantin-Teodosiu D, Laithwaite D, Simpson EJ & Greenhaff PL (2006a). I*nsulin stimulates L-carnitine accumulation in human skeletal muscle*. FASEBJ 20, 377–379.
7. Capaldo, B., et al., *Carnitine improves peripheral glucose disposal in non-insulin-dependent diabetic patients.* Diabetes research and clinical practice, 1991. 14(3): p. 191-5.
8. Schroeder, M.A., et al., *The cycling of acetyl-coenzyme A through acetylcarnitine buffers cardiac substrate supply: a hyperpolarized 13C magnetic resonance study.* Circulation. Cardiovascular imaging, 2012. 5(2): p. 201-9.
9. Muoio, D.M., et al., *Muscle-specific deletion of carnitine acetyltransferase compromises glucose tolerance and metabolic flexibility.* Cell metabolism, 2012. 15(5): p. 764-77.
10. Lindeboom L, Nabuurs CI, Hoeks J, Brouwers B, Phielix E, Kooi ME, Hesselink MK, Wildberger JE, Stevens RD, Koves T, Muoio DM, Schrauwen P, Schrauwen-Hinderling VB. The Journal of clinical investigation 2014;124:4915-4925
11. Le, D.S., et al., *Repeatability and reproducibility of the hyperinsulinemic-euglycemic clamp and the tracer dilution technique in a controlled inpatient setting.* Metabolism: clinical and experimental, 2009. 58(3): p. 304-10.
12. DeFronzo, R.A., J.D. Tobin, and R. Andres, *Glucose clamp technique: a method for quantifying insulin secretion and resistance.* Am J Physiol, 1979. 237(3): p. E214-23.
13. Thomsen, C., et al., *The day-to-day variation in insulin sensitivity in non-insulin-dependent diabetes mellitus patients assessed by the hyperinsulinemic-euglycemic clamp method.* Metabolism: clinical and experimental, 1997. 46(4): p. 374-6.
14. Mingrone, G., et al., *L-carnitine improves glucose disposal in type 2 diabetic patients.* Journal of the American College of Nutrition, 1999. 18(1): p. 77-82.
15. DeFronzo RA, Tobin JD, Andres R. Glucose clamp technique: a method for quantifying insulin secretion and resistance. Am J Physiol. 1979;237(3):E214-23. 11
16. Bergstrom, J., et al., *Diet, muscle glycogen and physical performance.* Acta Physiol Scand, 1967. 71(2): p. 140-50.

17. Ferranini, E., et al., *Interaction of carnitine with insulin-stimulatedglucose metabolisme in humans.* Am J Physiol, 1988. 255:946-52.

18. Szendroedi, J., et al. *Role of diacylglycerol activation of PKC in lipid induced muscle insulin resistance in human.* Proc Natl Acad Sci U S A., 2014: 9597-602.

19. Chows, L.S., *Training status diverges muscle diacylglycerol accumulation during free fatty acid elevation.* Am J Physiol Endocrinol Metab. 2014 Jul 1;307(1):E124-31

20. Natali, A., *Effects of Acute Hypercarnitinemia During Increased Fatty Substrate Oxidation in Man.* Metabolism. 1993 May;42(5):594-600.

Appendix 1A: Randomisation intervention arms

Randomisation (Set=subject)	Visit 4	Visit 5	Visit 6
Set 1	3	2	1
Set 2	2	3	1
Set 3	1	3	2
Set 4	2	1	3
Set 5	1	2	3
Set 6	3	1	2
Set 7	1	2	3
Set 8	2	1	3
Set 9	2	3	1
Set 10	3	2	1
Set 11	3	1	2
Set 12	1	3	2
Set 13	2	1	3

Appendix 1B: Randomisation leg 1st muscle biopsy

Randomisation (Set=subject)	1st biopsy
Set 1	1
Set 2	1
Set 3	2
Set 4	2
Set 5	2
Set 6	1
Set 7	2
Set 8	1
Set 9	1
Set 10	2
Set 11	2
Set 12	1
Set 13	1

Appendix 2A: Overview blood draws during hyperinsulinemic euglycemic clamp

Blood draw	Time point	Amount (mL)
Fasted	T=0	7
Baseline	T=90, 100, 110, 120	4 x 7 = 28
Insulin stimulated	T=450, 460, 470, 480	4 x 7 = 28
Additional samples	T=180, 240, 300, 360, 420	5 x 7 = 35
Glucose measurements	Every 5 minutes for 5.5 hours	66 x 1 = 66
“uitclampen”	Maximal 12 sample	12 x 1 = 12
**Total**		**176 mL**

Appendix 2B: Overview blood draws during entire study protocol

Visit	Measurement	Amount (mL)
Visit 1	Screening	10
Visit 2	Bod Pod + VO2max	0
Visit 3	^{1}H-MRS cycling test	0
Visit 4	Hyperinsulinemic euglycemic clamp	176
Visit 5	Hyperinsulinemic euglycemic clamp	176
Visit 6	Hyperinsulinemic euglycemic clamp	176
**Total**		**538 mL**
